# Glycosylation Biomarkers Associated with Age-Related Diseases and Current Methods for Glycan Analysis

**DOI:** 10.3390/ijms22115788

**Published:** 2021-05-28

**Authors:** Beatrix Paton, Manuel Suarez, Pol Herrero, Núria Canela

**Affiliations:** 1 Eurecat, Centre Tecnològic de Catalunya, Centre for Omic Sciences, Joint Unit Eurecat-Universitat Rovira i Virgili, Unique Scientific and Technical Infrastructure (ICTS), 43204 Reus, Spain; beatrix.paton@eurecat.org (B.P.); nuria.canela@eurecat.org (N.C.); 2 Nutrigenomics Research Group, Departament de Bioquímica i Biotecnologia, Universitat Rovira i Virgili, 43007 Tarragona, Spain

**Keywords:** ageing, glycosylation, *n*-glycan, age-related disease, glycan analysis, mass spectrometry

## Abstract

Ageing is a complex process which implies the accumulation of molecular, cellular and organ damage, leading to an increased vulnerability to disease. In Western societies, the increase in the elderly population, which is accompanied by ageing-associated pathologies such as cardiovascular and mental diseases, is becoming an increasing economic and social burden for governments. In order to prevent, treat and determine which subjects are more likely to develop these age-related diseases, predictive biomarkers are required. In this sense, some studies suggest that glycans have a potential role as disease biomarkers, as they modify the functions of proteins and take part in intra- and intercellular biological processes. As the glycome reflects the real-time status of these interactions, its characterisation can provide potential diagnostic and prognostic biomarkers for multifactorial diseases. This review gathers the alterations in protein glycosylation profiles that are associated with ageing and age-related diseases, such as cancer, type 2 diabetes mellitus, metabolic syndrome and several chronic inflammatory diseases. Furthermore, the review includes the available techniques for the determination and characterisation of glycans, such as liquid chromatography, electrophoresis, nuclear magnetic resonance and mass spectrometry.

## 1. Introduction

Protein glycosylation is the biochemical process for which a carbohydrate molecule is covalently attached to a protein functional group. In biology, glycosylation mainly refers to the enzymatic process that binds glycans to proteins, affecting intracellular processes like folding and transport, and playing an important role in many cellular signaling and communication events. The two most common types of protein glycosylation are *n*-linked and O-linked glycosylation. Regarding *n*-linked glycosylation, the glycan is added at the Asn residue on a nascent polypeptide within the consensus sequence Asn-X-Ser/Thr, even though this does not occur at every potential glycosylation site. On the other hand, O-linked glycosylation can occur at any hydroxyl group of a Ser or Thr residue within the protein sequence [1,2], although very few sites are occupied [3].

Many studies suggest that glycans modify the functions of proteins and take part in intra- and intercellular biological processes. Most cell-surface and secreted proteins have glycans attached to them [4] that are involved in molecular recognition processes that occur in viral infections, cell adhesion in inflammation and tumour metastasis, amongst other events [2]. Furthermore, protein-glycan interactions are also known to play a role in many processes affecting disease progression [5]. For instance, aberrant glycosylation is often present in patients with cancer [6,7], diabetes and inflammation [8,9].

Diseases are also driven by many diverse factors, including genetic variants, epigenetic dysregulation and environmental influences. Glycosylation, among other post-translational modifications (PTMs), can reflect the real-time status of these complex interactions and can provide potential diagnostic and prognostic biomarkers for multifactorial diseases [8,10]. Glycosylation is known to be altered in the process of ageing. For example, modifications in protein *n*-glycosylation can lead to an increase in certain types of glycoforms or even the formation of new ones, after surpassing the age of 40 years [11]. For many years, ageing has been considered to be a physiological condition that favours the onset of many diseases. However, the relationship between ageing and these diseases is more complex, as they share basic mechanisms, such as metabolism derangement, macromolecular damage, epigenetic modifications or inflammation [2,12]. This relationship resulted in the introduction of the term age-related diseases.

Ageing is characterised by a progressive loss of physiological integrity, leading to impaired function and increased vulnerability to death [13] This process is partially modulated by genetic factors and non-genetic factors, including the nutritional habits of individuals. There is evidence indicating that dietary patterns play a central role and are recognised as major factors in the onset of chronic diseases, including cardiovascular diseases, diabetes and cancer. A better understanding of the interaction between nutrition and ageing is essential to unravel the mechanisms responsible for these positive/negative effects, to identify diet components promoting the quality of life in old age and to contribute to the prevention of late-life disabilities [14]. Few studies have shown how the dietary patterns in humans affect glycosylation, however there is evidence that diet may influence disease state by altering glycosylation [15].

The analysis of protein glycosylation is a challenging task due to the primary structure of glycans. Glycans are biopolymers composed of monosaccharides with many chiral centres that are connected by glyosidic linkages [16]. They can have complex three-dimensional structures and the function of the glycans can be considerably influenced by their stereoisomerism [17]. The structural complexity of glycans is due to their variable composition, linkage, branching and anomericity of the constituent monosaccharides, in combination with the general heterogeneity caused by the indirect control of their biosynthesis [18]. Consequently, the combination of different techniques is often necessary to determine the structure of a glycan, either alone or in association with a protein. Amongst the available techniques, electrophoresis [19,20,21,22], liquid chromatography coupled to mass spectrometry (LC-MS) [7,23,24,25,26] and nuclear magnetic resonance (NMR) [27,28] are the most frequently used. Differently from *n*-glycans, the determination of O-glycans has advanced slower because these have less, or none, defined sequence patterns and arrangements [29,30] limiting the research studies focused on them.

The main objective of this review is to gather the alterations in glycosylation profiles that have been stated and associated with age-related diseases. This includes both *n*- and O- glycosylation events, although studies regarding O-glycosylation are limited. In addition, the most frequently used techniques for the determination and characterisation of protein glycosylation will also be briefly discussed.

## 2. Glycans Associated with Ageing and Age-Related Diseases

Age-related diseases share common mechanisms with natural ageing. These mechanisms include adaptation to stress, loss of proteostasis, stem cell exhaustion, metabolism derangement, macromolecular damage, epigenetic modifications and inflammation [31]. If age-related diseases are manifestations of accelerated ageing, it is urgent to identify markers capable of distinguishing between biological and chronological age, in order to identify subjects that are more likely to develop age-related diseases [12].

Glycomic alterations related to specific disease states such as cancer [10], inflammation [32], neuronal diseases [33] and diabetes [34] have been reviewed elsewhere, however, less information about glycomic changes associated with age and ageing-related diseases has been published—Miura and Endo have reviewed up until 2016 [2]. Some articles focus on disorders that induce or hasten the ageing process, such as Alzheimer’s disease (AD) [35] or posttraumatic stress disorder in which stress exposure can influence the immune system and accelerate cellular ageing [36]. These alterations can affect the *n*-glycan profile as well as the metabolome in animals and humans [36]. Data on glycan-based biomarkers used for diagnosis of diabetes, cancers and other complex diseases have been gathered in a review by Hu et al. [37]. This review summarises the clinically used glycan and glycoprotein-based biomarkers as well as the potential serum/plasma-derived *n*- and O-linked glycans as new biomarkers. Similarly, Zhang et al. described and categorised into different groups the clinically used glycan-based biomarkers, specifying as well whether the median values of glycan-related biomarkers are increased or decreased in cancer and other diseases compared to that of healthy controls [38].

Plasma and serum are the most frequently used sample on studies that determine glycan alterations in age-related diseases. Approximately 20% of all plasma proteins are immunoglobulins, a group of glycoproteins involved in innate and adaptive immunity. Immunoglobulin G (IgG) is the most abundant immunoglobulin and is very frequently measured in the study of the *n*-glycome. It is involved in anti-inflammatory and pro-inflammatory responses throughout the body. These properties are modulated by the *n*-glycans that are attached to the conserved Asn 297 of both heavy chains in the fragment crystallisable (Fc) portion of IgG. By changing the conformation of the Fc portion and the affinity for a number of receptors and lectins, the *n*-glycans control the inflammatory properties of Immunoglobulin G (IgG) [39,40]. More than 30 IgG glycoforms have been identified in human serum [41,42]. The majority of Fc *n*-glycans in human IgGs are galactosylated: the neutral IgG glycome consists of a percentage of approximately 40% of neutral glycans without galactose, above 40% of neutral glycans with one terminal galactose and 20% of neutral glycans with two terminal galactoses [43]. Decreasing levels of galactosylated glycoforms have been directly linked to ageing and to immune activation [43,44]. 

Different glycomic methodologies are used depending on each research, leading to a low comparability between studies which does not allow to draw clear conclusions on whether there are plasma glycome signatures specific to a certain disease [45]. Only a few studies show the potential of plasma glycans in differential diagnosis on larger sample sets, as stated by Dotz and Wuhrer [45].

The following sections summarise the existing glycan alterations found in recent studies focused on the determination of the glycosylation profile in ageing and several age-related diseases.

### 2.1. Chronological Ageing

Ageing is a complex process which implies the accumulation of molecular, cellular and organ damage, leading to a loss of function and increased vulnerability to disease and eventually to death [46]. The determination of molecular markers of age is of special interest in order to be able to predict, monitor and provide insight into age-associated physiological decline and disease [47]. Table 1 shows some of the reported glycan alterations related to chronological ageing.

Three major *n*-glycan structures present in human blood glycoproteins (serum, plasma and immunoglobulins fraction) have shown clear changes with ageing. Agalactosyl *n*-linked oligosaccharides (NG0A2F and NG0A2FB) increase with age whereas core-fucosylated biantennary *n*-glycans (NA2F) decrease with age [22]. A similar trend was observed in a study which aimed to evaluate the effects of the age and gender on the human serum *n*-glycans profiles: NGA2F and NGA2FB increased gradually with ageing whereas NA2F decreased [59]. Additionally, before the age of 50 years these three glycans changed only slightly with age, but the difference between age groups 41–50 and 51–60 years was statistically significant, indicating that the age-related physiological changes occurred in the fifties [59]. These findings suggest that galactosylation and fucosylation of human sera *n*-linked oligosaccharides are considered to be an age-related molecular parameter [22]. More specifically, galactosylation tends to decrease with ageing, as digalactosylated glycopeptides are less abundant in older individuals. Additionally, nongalactosylated glycopeptides are more abundant in older individuals and decreased levels of nongalactosylated glycopeptides from IgG containing a bisecting GlcNAc are considered an early feature of familial longevity detectable at middle age [56].

**Figure 1:**
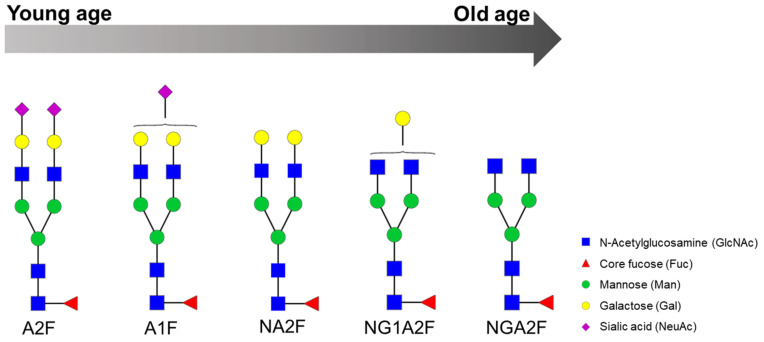
Decrease of galactosylated and sialylated structures with age (disialogalactosylated core-α-1,6-fucosylated biantennary, A2F; sialogalactosylated core-α-1,6-fucosylated biantennary, A1F; bigalactosylated core-α-1,6-fucosylated biantennary, NA2F; galactosylated core-α-1,6-fucosylated biantennary, NG1A2F; agalactosylated core-α-1,6-fucosylated biantennary, NGA2F).

As well as galactosylation, sialylation also decreases with age in the adult population, showing significant sex dependence. Furthermore, females around the age of 45 to 60 years show the most prominent drop in the levels of galactosylated and sialylated glycoforms, which is the same age in which females usually enter menopause. [55] In younger individuals, the incidence of bisecting *n*-acetylglucosamine is increased and reaches a plateau at older age [55]. Differently, in children, Fc galactosylation remains relatively constant with age, while bisection increases and fucosylation and sialylation decrease [60]. Figure 1 displays the general trends observed in the aforementioned studies regarding glycosylation associated with ageing.

An ageing biomarker named GlycoAgeTest has been developed, which could possibly forecast disease progression during ageing. This marker is the log of the ratio of two glycans (NGA2F and NA2F), which remains steady up to the age of 40 years and thereafter gradually increases to reach its highest level in nonagenarians, as stated by Vanhooren et al. [11]. Furthermore, patients with dementia or Cockayne syndrome have shown to have a higher GlycoAgeTest level than age-matched healthy individuals. They concluded that the value of GlycoAgeTest is better than chronological age for estimating the physiological age of a human individual, and that it could be used as an ageing biomarker for healthy humans [11].

Several studies have focused on determining specific proteins that might show variations of glycosylation with age. For instance, advancing age has been associated with changes in IgG glycosylation, inducing pro-inflammatory modifications in this glycoprotein [48,49]. These modifications are capable of fuelling an inflammatory vicious loop and may induce apoptosis of cells in surrounding tissues [32]. Furthermore, glycosylation patterns of α1-antitrypsin (αAT) enriched fractions have been found to be associated with chronological age. Moreover, several glycans in the αAT enriched fraction are associated with physiological parameters marking cardiovascular and metabolic diseases [58]. Other examples of proteins that differ with age are protein C, plasminogen [61,62] and α-2-macroglobulin (α2M) [51]. Specifically, α2M is a glycosylated broad-spectrum inhibitor of numerous proteases, including those involved in blood coagulation. Glycosylation characteristics can affect protein structure and function. This fact was evaluated in a study that compared glycosylation characteristics of α2M in newborn umbilical cord (NUCP) and adult pooled plasmas. No significant difference was reported in *n*-glycan macroheterogeneity between α2M obtained from NUCP and adult plasma. Nevertheless, differences were observed in glycan microheterogeneity, with an increased sialic acid content released from NUCP α2M [51]. In addition, glycans attached to α2M have been analysed in a human population of different ages, obtaining increased levels of α2,6-linked sialic acid, mannose, *n*-acetylglucosamine and multiantennary complex type *n*-glycans [50].

Other specific proteins of interest for their *n*-glycan composition are often determined. For instance, *n*-glycan composition of circulating molecules from the glycoprotein hormone thyrotropin (TSH) have been characterised in young children [53]. The TSH molecules are post-translationally modified in the Golgi apparatus within the cell where the branching of the *n*-glycans and their decoration with the terminal anionic monosaccharide residues occurs. TSH molecules modulate the biological properties of TSH in different physiological and clinical situations. Compared to adults, children have shown a lower degree of *n*-glycosylation and the lowest levels of sialylation have been determined in younger children [53].

Most studies determine the *n*-glycome in plasma, which is characteristic for a significant decrease of complex *n*-glycans observed with ageing. Differently, in skin glycomics, the epidermis has an abundance of high mannose *n*-glycosylated proteins, which play a role in stratum corneum lipid remodelling, desquamation and barrier function. Significant quantitative decreases have been observed in high mannose glycans in the stratum corneum from aged skin compared to young skin [54].

*n*-glycans have also been measured in the gut microbiomes of patients, showing that the proportion of various types of *n*-glycan biosynthesis is higher in the gut microbiomes of centenarians than in the elderly and adults. Therefore, *n*-glycan biosynthesis can be associated with the stability of gut microbiome in centenarians [63].

### 2.2. Neurodegenerative Diseases

Neurodegenerative diseases are characterised by the functional loss and death of neurons, leading to symptoms that affect the daily lives of patients [64]. Glycan alterations have been observed in several neurodegenerative diseases, such as AD, amyotrophic lateral sclerosis (ALS) or Parkinson’s disease (PD). AD is a common age-related chronic neurodegenerative disease [65] characterised by its cognitive impairment [2]. Several alterations are related to the development of this disease, such as the increase in brain deposits of aggregated amyloid-β and hyperphosphorylated tau proteins [66] or the upregulation of receptors for advanced glycation end product, which is the primary mechanism by which amyloid-β traverses the blood-brain barrier [67]. Considering that altered immune responses in AD are not only related to brain but also to peripheral blood, the measurement of IgG Fc glycosylation could be potentially used as a clinical marker of inflammation and/or impaired immune responses for monitoring AD progression [24]. Table 2 shows select examples of glycan alterations related to several neurodegenerative diseases.

Individuals with AD show an elevated proinflammatory activity in blood, shown through increased levels of inflammation mediating proteins and pro-inflammatory IgG Fc glycoforms [24]. Low abundance of complex galactosylated and sialylated forms in AD has been found, as well as differences between genders. Prior to the AD onset, declining galactosylation observed in females was inversed in males. This may indicate that females could have a lower ability to suppress peripheral inflammation compared to male patients [24]. Further studies need to address the pathological meaning of protein glycosylation changes in AD patients.

The glycosylation of collapsin response mediator protein 2 (CRMP-2) has been shown to be reduced in AD, while the glycosylation of glial fibrillary acidic protein is increased. CRMP-2 regulates the assembly and polymerisation of microtubules and is associated with neurofibrillary tangles in AD. These aberrant glycosylations in AD may help understand the mechanisms of neurodegenerative diseases [72].

ALS is another fatal disease characterised by the selective loss of motor neurons in the spinal cord, brain stem and motor cortex which leads to progressive muscle weakness, paralysis, respiratory muscle failure and death, usually within 2–5 years [73]. Neuroinflammation occurs in ALS, implying the activation of glial cells and infiltration of peripheral immune cells [73]. High levels of sialylated glycans and low levels of core fucosylated glycans have been measured in serum derived *n*-glycans of patients with ALS, compared to healthy volunteer sera. A distinct glycan was measured in all ALS patients, A2BG2, a galactosylated structure with a bisecting GlcNAc lacking the core fucose. This glycan increases the affinity of IgG to the activating receptor CD16 on effector cells, consequently enhancing Antibody-Dependent Cellular Cytotoxicity (ADCC). These results suggest that glycans of ALS-IgG may serve as a biomarker for the disease and may be involved in neuronal damage [68].

The IgG *n*-glycosylation profile has also been measured in cerebrospinal fluid of ALS patients. Diantennary *n*-glycans predominantly with proximal fucose and some bisecting GlcNAc; agalacto-, mono- and digalactosylated as well as α2,6-sialylated structures have been detected [69,70]. The same study established the Gal-Index parameter, a representation of the levels of galactosylated *n*-glycans, which could be further explored as a potential ALS biomarker [69]. Furthermore, increased levels of galactosylated structures [69] and monosialylated diantennary glycans A2G2S(6)1 and FA2G2S(3)1 have been measured in ALS patients [70]. A useful future approach would be to measure IgG galactosylation in blood samples (either serum or plasma) in order to explore differences between ALS patients and healthy population [69].

Similar to AD, altered sialylation has been determined in PD patients. Specifically, low sialylation on tri-antennary glycans with 2 and 3 terminal sialic acids and increased fucosylation have been measured [71]. Furthermore, as patients with PD age, the propensity to become more pro-inflammatory increases faster than what would be expected [40].

### 2.3. Cancer

There is a current interest in determining glycoproteins, and their corresponding glycans, which have critical importance in cancer. No general cancer-associated *n*-glycans currently exist but all of tetraantennary *n*-glycans in cancer patient sera are increased except in pancreatic cancer, indicating that increased tetraantennary *n*-glycans are a common *n*-glycan structural feature in different types of cancer. However, no comparable pattern of *n*-glycan profiles is observed among other cancers, reaching the conclusion that each type of cancer is unique and aberrant *n*-glycans in serum could help distinguish different classes of cancer [37]. In Table 3, several glycan alterations reported in cancer are displayed.

Fucosylation is one of the most important types of glycosylation in cancer and it regulates the biological functions of adhesion molecules and growth factor receptors. Therefore, it is thought that changes in fucosylation could provide a novel strategy for cancer therapy [89].

For instance, significant changes in high-mannose and fucosylated biantennary complex *n*-glycans have been observed in the serum of prostate cancer patients [86], as shown in Table 3. Similarly, preliminary data states that there are pronounced age-associated changes at the level of fucosylation and branching of glycans in transferrin, as well as an increased degree of fucosylation in colorectal carcinoma [82]. These findings are consistent with previous studies in which colorectal cancer patients had dramatically higher levels of sialylation and fucosylation [83]. The changes in fucosylation levels are also present in breast cancer, where increased levels of high-mannose and core-fucosylated glycans, as well as decreased levels of bisected and sialylated glycans were detected on breast cancer tissues compared to para-carcinoma tissues. Furthermore, six *n*-glycans have been associated with breast cancer and three high-mannose *n*-glycans (F0H6N2S0, F0H7N2S0, F0H8N2S0) have been shown to exhibit significant diagnostic accuracy in both breast cancer tissues and cells. Moreover, a negative correlation between sialylated glycans and the age of patients has been identified [90].

The glycosylation state of various types of cancer during the progression or evolution of the disease has also been stated. For instance, the serum *n*-glycan profile has claimed to be altered during the development of gastric cancer in a study where nine *n*-glycan structures were compared. The abundance of individual *n*-glycan structures was analysed, determining that the abundance of core-fucosylated structures decreased significantly in gastric cancer, similar to what occurs in colorectal cancer [84]. This finding could aid with the diagnose of gastric cancer in its early stages and further studies focused on the changes in glycosylation in each step of cancer development and progression could improve the diagnosis, monitoring and screening of gastric cancer [79].

*n*-glycans could have a potential diagnostic ability in breast cancer, improving the understanding of its underlying molecular and cellular mechanisms [90]. More specifically, targeted blood plasma glycomics is thought to be a promising source of noninvasively diagnostic and prognostic biomarkers for lung cancer. Several glycan features have been quantified and distinguished from controls, predicting survival in patients at all stages, including 2-linked mannose, α2−6 sialylation, β1−4 branching, β1−6 branching, 4-linked GlcNAc and antennary fucosylation. Furthermore, most of these identified glycans are independent of smoking status, age, gender and histological subtypes of lung cancer [81].

A limitation in most studies focused on the study of plasma or serum glycosylation is to assess if the altered glycans are either from glycoproteins made in the liver, from immunoglobulins made by immune cells or products from cancer cells. In order to address this issue, Hu et al. purified several serum glycoproteins that are not produced by cancer cells from both healthy controls and different types of cancer patients [37]. One of these glycoproteins is IgG, which is the most prevalent serum immunoglobulin with concentrations of approximately 10–15 mg/mL. IgG is produced by B lymphocytes but not from cancer cells. A total of 36 *n*-glycan structures have been characterised from IgG purified from the sera of gastric, ovarian, breast and lung cancer patients, exhibiting different *n*-glycan structures compared to cancer-free patients [37]. Another study focused on obtaining a detailed profile of IgG *n*-glycans in breast cancer patients, claimed that *n*-glycan structures of IgG in the sera of breast cancer patients had better sensitivity and specificity compared to cancer biomarkers used in the clinical area [26]. Most importantly, serum IgG *n*-glycans of stage 0 breast cancer patients are already different from cancer-free controls, indicating that cancer might be accompanied by abnormal B lymphocyte-produced *n*-glycans of IgGs from the earliest stages of the disease [37].

Haptoglobin is another relevant glycoprotein found to be a biomarker for different cancers, including esophageal, gastric, colon, gallbladder, pancreatic, prostate and ovarian. This protein is produced in the liver and contains four glycosylation sites (Asn 184, Asn 207, Asn 211 and Asn 241). All its biantennary *n*-glycans have been reported to be increased, triantennary *n*-glycans without core fucose are decreased, and core-fucosylated glycans are increased in the sera of gastric, ovarian and pancreatic cancer patients [37]. A study focused on the characterisation of fucosylation on haptoglobin among different cancer types, showed that relevant differences in the structure or fucosylation of *n*-glycan were not detected in esophageal, gastric, colon, gallbladder, pancreatic and prostate cancers, suggesting that haptoglobin in the sera of cancer patients might be produced in the liver [88]. These results suggest that glycan structures in liver are similarly affected when different types of cancer are present [37].

Glycans attached to α2M are also of interest since it is a glycoprotein associated with chronic inflammation. Previous reports stated that α2M isolated from patients with colorectal cancer contained more α2,6Sia, GlcNAc and mannose residues, as well as more multiantennary complex type *n*-glycans, compared to α2M isolated from healthy participants [85].

Many other *n*-glycosylations have been associated with other types of cancer, such as the presence of bisecting *n*-acetyl-glucosamine *n*-glycans, increased levels of α2-6 sialylated and *n*,N’-diacetyl-lactosamine *n*-glycans, which have been identified as characteristic glycan features that are unique to ovarian cancer membrane proteins [7] or the abundance of β1,6-branched oligosaccharides which is found in breast carcinoma nodal metastasis [6].

### 2.4. Type 2 Diabetes Mellitus

New evidence of glycosylation diversity in Type 2 diabetes mellitus (T2DM) has been found, where IgG glycan traits have been associated with this disease, reflecting an increased proinflammatory and biological ageing status. These associations are in concordance with a decrease of agaloctosylated glycans without galactose and an increase of monogalactosylated glycans and fucosylated structures with bisecting GlcNAc [8]. These results show that lower levels of agalactosylation could increase the risk of T2DM, which matches with previous results on T2DM [9]. A few selected glycan alterations associated with T2DM are shown in Table 4. 

The following particular *n*-glycans have been found to be significantly reduced in T2DM patients: α(1,6)-linked arm monogalactosylated and core-fucosylated diantennary *n*-glycans (NG1A2F) [19]. This tendency is also present in other studies where T2DM patients have shown decreased core fucosylated glycans and significantly decreased levels of low-branching plasma *n*-glycans. On the other hand, T2DM patients also show increased levels of high branching plasma *n*-glycans as well as statistically significantly increased levels of di (S2) and trisialylated (S3) *n*-glycans.

Galactosylation has also been associated with ageing in both controls and T2DM cases [91]. When correlating *n*-glycans and age in T2DM, levels of FA2G2 and FA2BG2 (both neutral glycans) decreased with age, whereas A2[3] BG1S[3]1 (monosialylated glycan) [91] increased with age. These findings are consistent with previous reports, where significant correlations were found between plasma *n*-glycans and age [9,40,49]. In addition, increased levels of fucosylated bisecting GlcNac have also been identified in studies of biological age in distinct populations [48,49], suggesting that this tendency in T2DM patients may be a chronic inflammatory condition as well as a biological ageing condition [8].

Some specific glycosylation changes are reflective of inflammation, such as increased α2,6-linked sialylation. Nevertheless, a study determined thatα2,3-linked sialylation decreased in T2DM patients, which differs from reports on acute and chronic inflammation [92]. This study also found that levels of α2,3-linked and α2,6-linked sialylation fucosylated glycans increased with age, whereas non-fucosylated glycans decreased with age.

### 2.5. Metabolic Syndrome and Related Diseases

Metabolic syndrome (MetS) is a cluster of metabolic abnormalities that includes hypertension, central obesity, insulin resistance and atherogenic dyslipidemia. MetS is strongly associated with an increased risk of developing atherosclerotic Cardiovascular disease (CVD) and even though characteristics of the MetS occur in some children and adolescents, the prevalence of MetS increases with age [94]. Table 5 shows reported glycan alterations associated with several MetS and related diseases.

Significantly elevated levels of NGA2FB and NA3F have been determined in women with MetS, while the level of the α(1,6)-arm monogalactosylated glycan (NG1A2F) was significantly lower in women with MetS compared to their healthier peers. The effect of physical exercise was also tested in patients with MetS, resulting in increased levels of NGA2FB and NA3F and lower levels of NG1A2F compared to those without MetS. This study confirmed the existence of *n*-glycans linked to metabolic risk in older adults. Furthermore, the results indicated that physical activity is related to a specific *n*-glycan profile despite the metabolic risk status [20]. Previous reports have similar changes in levels of *n*-glycans, such as those reported by Testa et al., claiming increased levels of NGA2FB and NA3F and decreased levels of NG1A2F in older women with MetS [19]. Moreover, reduced levels of NA3 and NA4 have been associated with the adherence to the physical activity [20], which is an alteration that has already been associated with metabolic abnormalities [19]

*n*-glycans have also been studied in specific populations. For instance, a research focused on the correlation between *n*-glycan structures and MetS components in Chinese Han individuals and Caucasian individuals from the Croatian island Korčula. The most prominent observation was the consistent positive correlations between different forms of triantennary glycans and negative correlations between glycans containing core-fucose with MetS components in both populations. The successful replication of these observations in the population of a Croatian island indicates that *n*-glycans of human plasma could reliably reflect alterations of human metabolism and could be potential biomarkers of MetS [95].

Metabolic syndrome is associated with an increased risk for CVD [102], which results in 40% of all deaths and ranks as the leading cause in population aged 65 or older [103]. As well as metabolic syndrome, other conditions such as insulin resistance, MetS and prediabetes can lead to a set of metabolic abnormalities (cardiometabolic disease) which can eventually lead to CVD [21]. Several risk factors such as abdominal obesity, dyslipidemia, hyperglycemia and hypertension are related to cardiometabolic disease [104]. Previous reports have shown that IgG *n*-glycans are associated with some of these risk factors, including hypertension [96,97,98], T2DM [9,25] and dyslipidemia [101].

IgG glycosylation traits have been correlated with the atherosclerotic CVD risk score determined by the GlycA test. This test measures plasma glycoprotein acetylation by Nuclear Magnetic Resonance (NMR) and is able to predict higher cardiovascular risks by reflecting inflammatory pathways. The data obtained has comprehensive measures of protein glycosylation, which highlights the potential value of glycomics in identifying such pathways of disease, the reproducibility of results across different cohorts and the extent to which CVD risk can be captured by these measures [56]. Even though some IgG glycans have been reported to be associated with higher CVD risk, others are associated with lower CVD risk. Specifically, glycans that contain three exposed GlcNAcs or glycans that contain both bisecting GlcNAc and one sialic acid, are positively associated with CVD risk (consistent with the previous GlycA reports), while sialylated glycans without a bisecting GlcNAc are negatively associated. Furthermore, increased levels of non-galactosylated glycoforms with a bisecting GlcNac are reported to associate with higher age while decreased levels are associated with longevity [56]. However, results regarding the association between glycosylation traits and CVD risk showed that this association was not dependent on the presence of other sugar residues, as agalactosylated, monogalactosylated and sialylated *n*-glycans with a bisecting GlcNAc were positively associated with CVD risk [99].

Age and T2D are factors included in the 10-year ASCVD cardiovascular risk score assessment and have a positive association with a bisecting GlcNAc that increases ADCC mediated by the binding of the antibody to the Fcγ-receptor, which is regulated by IgG glycosylation [105]. ADCC is increased by the same glycan traits that are involved in the pro-inflammatory pathway and inflammation is known to be the underlying mechanism of CVD’s development [106]. Differently to bisecting GlcNAc which is related to pro-inflammatory activity, sialylation and core-fucosylation are consistently associated with anti-inflammatory activity [107]. For instance, a core-fucosylated digalactosylated monosialylated glycan remains strongly associated with the 10-year ASCVD risk score and has been found to be strongly negatively correlated with VLDL levels [99]. VLDL itself is a risk factor for CVD being associated with hypertriglyceridemia and dyslipidemia in general [108]. Further studies are needed which focus on the role of glycosylated structures in predicting cardiovascular events, and in particular their interaction with VLDL [99].

Hypertension is also a risk factor associated with MetS. It is a prevalent condition with numerous health risks, and its incidence is greatest among older adults [109]. *n*-glycosylation alteration of IgG has been associated with blood pressure status as galactosylation has been found to decrease with increasing blood pressure [96], consistent with results from Wang et al. [97]. These exact same associations have been made in studies about inflammatory bowel disease (IBD) [110] and AD [24]. Individuals with AD manifest an elevated proinflammatory activity in blood, shown through increased levels of inflammation mediating proteins and pro-inflammatory IgG Fc glycoforms. Moreover, the inflammatory status in the periphery is known to be different depending on the gender [24]. Therefore, robust associations of subclass-specific IgG Fc *n*-glycosylation profiles may become informative biomarker indicatives of the proinflammatory and biological state induced by hypertension [98].

As well as hypertension, dyslipidemia is also a MetS risk factor. In dyslipidemia patients, blood lipids have been associated with the loss of galactose and sialic acid, as well as the addition of bisecting GlcNAcs, which is thought to be related to the chronic inflammation accompanying the development of dyslipidaemia. Furthermore, due to the alterations of IgG *n*-glycosylation profiles, they are considered to be potential biomarkers for dyslipidemia [101].

A few of the aforementioned risk factors and conditions, such as diabetes, hypertension and ageing can induce the development of chronic kidney disease [100,111]. Fourteen glycan traits with galactosylation, sialylation and bisecting *n*-acetylglucosamine features have been associated with renal damage, showing the role of IgG glycosylation in kidney function [100]. Moreover, this finding provides novel insight into the pathophysiology of chronic kidney disease as well as potential diagnostic and therapeutic targets [100].

### 2.6. Chronic Inflammatory Diseases

Glycan composition is altered in patients suffering from acute and chronic inflammatory diseases, such as IBD, RA and idiopathic inflammatory myopathies (IIM). A select amount of glycan alterations associated with these diseases are shown in Table 6. Changes in glycosylation have been found in IBD, a term used to define a group of inflammatory conditions of the colon and small intestine, which is divided into two forms, Crohn’s disease (CD) and ulcerative colitis (UC). Patients with CD or UC show lower levels of IgG galactosylation than controls [23] which has also been observed in other studies focused on IBD [110]. More specifically, plasma samples from patients with IBD have been found to have a higher abundance of large-size glycans compared with controls, a decreased relative abundance of hybrid and high-mannose structures, lower fucosylation and galactosylation and higher sialylation (α2,3- and α2,6-linked). Patients with CD can be differentiated from UC patients due to a higher bisection, lower galactosylation and higher sialylation (α2,3-linked) [112]. To our knowledge, this same study was also the first to present novel associations of sialic acid linkages with age for both healthy population and IBD patients [112]. The linkage of sialic acids, which can be of α2,3- or α2,6- type, is reported to affect various biological processes, such as the immune response in tumour cells [113]. Therefore, sialic acid linkages in the context of IBD were evaluated in order to provide insights into disease mechanisms and help the development of targeted treatment strategies [114]. Compared with healthy population, general sialylation was found to be significantly increased in UC, and even more in CD patients. CD and UC patients had higher relative levels of both α2,3- and α2,6- linked sialic acids compared with healthy controls in all traits, except for the α2,6-sialylation of tetra-antennary structures, which was lower in IBD. The strongest association distinguishing UC from CD was found in the sialylation per galactose in diantennary fucosylated glycans [112]. Patients with IBD share several immunologic similarities with rheumatoid arthritis (RA) patients. As the latter ones have increased levels of serum agalactosyl IgG, a study focused on measuring the oligosaccharide structure of serum IgG in patients with IBD. Results showed that the agalactosyl fraction of the fucosylated IgG oligosaccharides was significantly greater in IBD patients compared to healthy volunteers. Most importantly, the extent of agalactosylation of IgG correlated with disease activity of IBD and is a potentially effective diagnostic marker for IBD [115].

RA is another chronic autoimmune, inflammatory disease, which affects mainly the diarthrodial joints [124]. The disease affects about 1% of white population and can occur at any age. Nevertheless, most patients are between 40–70 years old [116]. Glycosylation, citrullination and carbamylation PTMs have been strongly associated with RA. The role of these PTMs in the pathogenesis of RA has been reviewed by Mastrangelo et al. [125]. Moreover, recent advances in glycan analysis for RA cases have also been reviewed [126]. The aim to perform glycomic analyses in RA patients has been led by the lack of robust diagnostics of RA and its remission status. A study reported that the presence of two agalactosylated glycans (FA1 and A2) and a di-sialylated, galactosylated biantennary glycan (FA2BG2S2) increased the likelihood of occurrence of RA, whereas the presence of glycan A2BG2S2 (digalactosylated and disialylated glycan) indicated a low chance of occurrence of the disease [121]. It has been well documented that RA patients exhibit decreased galactosylation of its conservative *n*-glycans (Asn 297) in CH2 domains of the heavy chains. This decrease of galactosylation is proportional to disease severity [116] and is correlated with the risk of developing the disease [116,117,126,127].

In order to improve RA treatment, the effectiveness of the treatment has been correlated with IgG galactosylation. In this approach, the use of therapeutic agents such as methotrexate and remicade indicated the improvement of IgG galactosylation during therapy of RA patients [116]. Another study showed that IgG *n*-glycosylation correlates with RA years before RA diagnosis and indicates that *n*-glycans are involved actively in the disease pathology [117]. These findings correlate with another study which reported evidence on dysregulated humoral immunity in RA by examining aberrant glycosylation of IgG. This dysregulation began at least 3.5 years prior to onset of symptoms. Findings suggest that aberrant IgG galactosylation substantially predates onset of arthritis and the diagnosis of RA, and resides selectively in the anti-citrullinated peptide autoantibody fraction [119]. These early changes in the *n*-glycome could be useful for the creation of a specific test for early diagnosis of RA, as well as being adequate for the evaluation of disease progression, remission and proper treatment [117]. Furthermore, galactosylation of IgG in patients with rheumatoid arthritis has been correlated with severity and duration of illness. A significant decrease of galactose ratio has been observed in patients with long duration of RA (more than 15 years) compared to patients who have had arthritis for less than 5 years. Moreover, decreased galactosylation of IgG is observed in RA patients, which correlates with severity and duration of RA and could be used in monitoring the progression in early arthritis [120].

IIM are chronic, autoimmune conditions characterised by weakness and inflammation of skeletal muscle. Extra-muscular organs, such as skin, joints and lung, are usually affected. Interstitial lung disease often occurs, which is a major cause of morbidity and mortality [123,128]. Autoantibodies are frequently found in patients with IIM, the most common being anti-Jo1 antibodies targeting histidyl transfer RNA synthetase (HisRS or Jo1). As IgG Fc-glycans affect IgG function and are altered in autoimmune diseases and autoantibodies, a study hypothesised that the total-IgG Fc-glycans from Jo1+ versus Jo1− patients and anti-Jo1-IgG would show characteristic differences, and that particular Fc-glycan features would be associated with specific clinical manifestations [123]. It was confirmed that the Fc-glycan profile of IgG1 in IIM patients contained less galactosylated epitopes compared to healthy controls, which is a feature associated with pro-inflammation. Moreover, the lower abundance in bisected and afucosylated forms was linked to anti-Jo1 autoimmune IgG [123].

## 3. Glycomics Techniques

As reviewed, protein glycosylation is a fundamental process that controls essential biological pathways. Therefore, the analysis of glycans is ultimately important. Several methods are available for the high-throughput separation and analysis of glycans. However, the low abundance of glycans hinders their identification, as well as the structural elucidation and quantitation of the glycome. Hence, pre-treatment of the sample is required to successfully characterise the full glycome. Regarding the determination techniques, a broad range of analytical platforms can be applied for the profiling, characterisation and analysis of glycans, being the most widely used liquid chromatography coupled with fluorescence detection (LC-FLR) [9,48,97,100], lectin-based microarray [50,82,85], capillary electrophoresis (CE) [58,71,118], matrix-assisted laser desorption/ionisation mass spectrometry (MALDI-MS) [52,75,92,112], LC-MS [23,24,49,96], DNA sequencer-aided fluorophore-assisted carbohydrate electrophoresis (DSA-FACE) [19,22,74,79], ion mobility (IM) and NMR [93]. Several reviews focus exclusively on describing the available techniques used for glycan analysis [129,130,131]. This review summarises the most commonly used techniques, focusing only on the determination of *n*-glycans, as O-glycans are less documented. Table 1 to 6 briefly describes the sample treatments and determination techniques used in several studies for the determination of *n*-glycans.

### 3.1. Sample Preparation

Reviews by Zhang et al. [132] and Xiao et al. [133] have specifically focused on describing the developments and advancements of sample preparation strategies for MS-based qualitative and quantitative *n*-glycans analysis. Briefly, these pre-treatment approaches include the release, separation, enrichment and derivatisation of *n*-glycans. 

The procedure starts with the detachment of *n*-glycans from the protein or peptide backbone. Two different approaches can be used for this step, a chemical release procedure or an enzymatic release procedure. The use of chemical release procedures, including hydrazinolysis [134] or β-elimination by alkaline borohydride [135] were mainly used in early studies of oligosaccharides. Nowadays, enzymatic methods are more commonly used [132]. Amongst the available endoglycosidases, PNGase F is the most frequently used for treating mammalian proteins. It cleaves the glycosidic bond between the side chain of the asparagine residue and the reducing end of an oligosaccharide, creating an aspartic acid residue and a free glycosylamine [132]. Endoglycosidases F1-F3 and H are also used in *n*-glycome studies. Briefly, endo F and H catalyse dissociation of the β-1,4-glycosidic bond formed between two GlcNAc units of the *n*-glycan core [136].

Extended digestion time is often needed due to the high complexity and wide dynamic range of glycoproteins within a biological system [132]. Therefore, several modifications to the deglycosylation procedure have been made in order to achieve a faster digestion: the deglycosylation of 15 μg of glycoproteins using an enzyme-friendly surfactant, RapiGest SF and a 50 °C incubation step, accelerates the PNGase F induced digestion, completing the process in 7 min [137]. Other approaches have been employed to accelerate the glycan release process, such as microwave-assisted digestion [138], high-pressure cycling reactor [139] and immobilised enzyme reactor [140].

Usually, a purification step or enrichment-oriented pretreatment is recommended to reduce the complexity of the sample and facilitate the determination of *n*-glycans [132] since the abundance of glycoprotein is low in many biological samples [132]. Moreover, some glycoforms reach down to the limit of detection of many analytical methods, hampering their identification and quantification [141]. For instance, lectin affinity chromatography is widely used in the field of isolation, fractionation and enrichment, due to the capacity of lectins to recognise and capture carbohydrates with certain motifs [142]. Other common enrichment methods are porous graphitised carbon, hydrophilic interaction chromatography (HILIC) and size exclusion capture [133].

### 3.2. Determination Techniques

#### 3.2.1. Fluorescence Detection

##### Lectin-Based Microarray

Lectin-based protein microarray is a method that enables the determination of released *n*-glycans in their native form. It has been reported that a very sensitive lectin-based protein microarray assay can be formulated and used to detect changes in glycan structures which accompany ageing or a disease [82]. Lectin-based microarrays are optimal for the determination of glycosylation changes and fits very well with biomarker research requirements [143], replacing conventional lectin-based analytical methods such as lectin blotting [85]. Moreover, they are also a useful tool for quantitative analysis of lectin-glycoprotein interactions.

Briefly, this technique consists of immobilising on a glass slide a panel of lectins (>20), for which specificity has been well documented. A fluorescent-labelled probe molecule is then added, which simultaneously interacts with the lectins. After performing extensive washing to remove the unbound probe, the resulting fluorescence (FLR) intensity on each lectin spot is measured, most commonly with a confocal-type FLR scanner [130,144]. By using this method, a fast analysis of a sample glycopattern can be obtained, rather than a precise identification of the glycan structures, which is the domain of Mass Spectrometry (MS) techniques [85].

##### Liquid Chromatography

LC is one of the mostly used separation techniques for the determination of glycans. It is commonly combined with FLR detection as its glycan quantification is considered to be better than the quantification that MS offers [145].

Specifically, HILIC is widely used for the separation of glycans. It was first introduced as a variant of normal phase chromatography in which analytes interact with a hydrophilic stationary phase. Analytes are retained on the hydrophilic stationary phase by hydrogen bonding, ionic interactions and dipole-dipole interactions [146]. HILIC differs from normal-phase chromatography because it uses polar aqueous mobile phases, eluting analytes in order of increasing hydrophilicity [147,148]. Accordingly, this technique is widely used for the analysis of very polar compounds [147], as hydrophilic compounds strongly retain on the stationary phase [146]. This chromatography is the most efficient separation method for native or reducing end labelled glycans which provides efficient separation of different isomers [147]. These advantages have permitted that HILIC-based enrichment techniques for glycans and glycopeptides become valuable tools in glycoproteomics [149]. This separation technique can be combined with FLR detection or MS [149]. Since larger glycans elute later (due to its large number hydrogen bonding groups), HILIC of glycans is sometimes known as “size fractionation”. As well as the size of the glycan, its charge and steric properties influence HILIC retention, resulting in the separation of isomers [149]. Often a single HILIC run may not be sufficient for structural assignment, hence additional anion exchange-LC and reversed phase liquid chromatography (RP-LC) separation steps may be used to obtain two- or three-dimensional mapping of glycans [150].

Reversed-phase chromatography is also widely used for the separation of glycans, as shown by Vreeker et al. by presenting an overview of the literature on reversed-phase separation of carbohydrates [16]. This technique is based on a noncovalent association between the nonpolar stationary phase and the nonpolar moieties of an analyte [16]. Most methods using reversed-phase columns are based on C18 separation. Characteristics can differ between columns, from column length, internal diameter and particle size which influence on the separation efficiency [16], to the density and nature of the nonpolar groups immobilised on the silica surface which influence the selectivity [151]. Oligosaccharides generally exhibit poor retention on C18-RP-LC, therefore derivatisation with a hydrophobic agent is required to allow their efficient separation. The chosen tag will not only influence the retention behaviour on the particular stationary phase, but also improve the sensitive and selective detection by UV absorption or FLR, as well as enhancing the ionisation and fragmentation behaviour in MS [18,152].

Two of the most used labelling reagents for Liquid Chromatography (LC) analysis are 2-aminobenzamide (2-AB) and procainamide [145]. Both labels use the same mechanism to bind to glycans: the primary amine group of the label reacts with the aldehyde group of the glycan, resulting in an imine, which will then be reduced to form a stable secondary amine. Using this approach, one molecule of label is stoichiometrically attached to one molecule of glycan, allowing the relative quantification of different glycans based on FLR intensity [153]. A third labelling compound is RapiFluor MS (RFMS). It uses a different binding chemistry that contains *n*-hydroxysuccinimide, which modifies the *n*-glycan glycosylamine, producing a *n*-glycan with a urea linked RFMS label. In addition, it contains a quinoline fluorophore for FLR detection and a basic tertiary amine to enhance positive mode electrospray ionisation (ESI) [145]. 6-aminoquinolyl-*n*-hydroxysuccinimidyl carbamate (AQC) uses a similar binding chemistry to RFMS, containing a *n*-hydroxysuccinimide carbamate and a quinoline fluorophore but lacking the basic tertiary amine. They react with both primary and secondary amines in aqueous solution and yields derivatives that are stable at room temperature for several days. Moreover, it has unique FLR properties which allow the LC-FLR analysis of derivatised samples without prior reagent removal and with minimal reagent interference [154,155].

##### Capillary Electrophoresis

CE is a high-resolution method that can separate complex carbohydrate molecules while offering excellent sensitivity. This method, in conjunction with laser-induced fluorescence (LIF) detection, can reach detection limits in the femtomolar range [156] and is optimal for profiling fluorophore-labelled oligosaccharides [157]. Briefly, this analytical system used for carbohydrate profiling separates and analyses oligosaccharides released from glycoproteins. Once the oligosaccharides have been released, the fluorophore 8-aminopyrene-1,3,6-trisulphonate (APTS) is used for labelling the oligosaccharides at their reducing terminus by reductive amination. Subsequently, the APTS derivatives of oligosaccharides are readily separated by CE [158]. Amongst other labels, APTS remains the method of choice for CE separations [159,160]. Glycan labelling with APTS through reductive amination is well-established and preferred over other strategies, such as hydraside or Michael addition [153].

CE provides fast and efficient separations, as well as a high degree of automation [158]. This technique is accessible to researchers through commercially available solutions to interface CE with MS, as well as improving the databases and software that will ease the data analysis [156]. By using instruments with 16-capillary arrays, exceptional throughput can be achieved compared to LC methods. Regarding the cost of the instrument, it is on par with LC and low-end MS approaches, and the cost of the sample is significantly low. The limitations of the technique include poor acceptance by the glycomics community and the lack of large structural databases [156].

Comparisons between various separation techniques have been carried out. It has been reported that CE and HILIC have similar performance and are both better suited to resolve complex mixtures compared to RP-LC [161]. Another study demonstrated that CE and HILIC showed comparable results, however electrophoresis was more cost-effective, consumed less sample and was operated with higher throughput [159].

Another electrophoresis-based technique is DSA-FACE, a high throughput technology platform that offers an accurate fingerprint of the glycan composition in serum, plasma and other body fluids [11,162]. This procedure requires only 2 μL of sample and involves the labelling of glycans, profiling and read-out [11]. It is robust, reproducible, sensitive and quantitative, as well as non-invasive. Furthermore, the method is easy and can be learned rapidly. None of the reagents are expensive and a DNA sequencer is the only specialised equipment required. Nevertheless, this technique does have a couple of limitations. Firstly, it misses information about the sialic acid distribution, as it does not profile desialylated *n*-glycans. Secondly, it lacks information about the sugar structures, which can be solved by carrying out exoglycosidase digestions to obtain more information about the structure of the glycan [162].

#### 3.2.2. Mass Spectrometry Detection

MS is becoming increasingly more important for structural determination and quantitative glycomic analysis due to its speed, simplicity, resolution and information content [163,164].

At present, MALDI and ESI are the most widely-used MS ionisation techniques for the analyses of carbohydrates [129,164]. In both MALDI and ESI, free glycans tend to be detected as metal (usually sodium) adducts in the positive ion mode, and as deprotonated or anion-adducted species in the negative-ion mode [129]. Even though native glycans can be analysed by MS, low sensitivity in positive mode MS and potential insource fragmentation leads to the inability to directly analyse native glycans [152]. To address this issue, several derivatisation methods have been used, such as reductive amination, permethylation and peracetylation [165]. Parameters of interest such as reproducibility and quantification can be measured using MS even though they can be compromised by ion suppression in complex samples, fluctuations in spectrometer performance or variability in matrix crystallisation for specifically MALDI-TOF MS applications [163].

Various mass analysers, available for the separation of ions in MS, are used in glycosylation studies. For instance, some of the most common are the quadrupole mass analyser [166], time of flight mass analyser [167], quadrupole ion trap mass analysers [168] and Fourier-transform ion cyclotron resonance [169,170]. Several parameters differ depending on the used mass analyser, such as the extent of glycan dissociation, which is lower in ion traps compared to quadrupole time-of-flight, triple quadrupole and Fourier Transform mass spectrometers with external dissociation cells [171].

##### Matrix-Assisted Laser Desorption/Ionisation Mass Spectrometry

**Figure 2:**
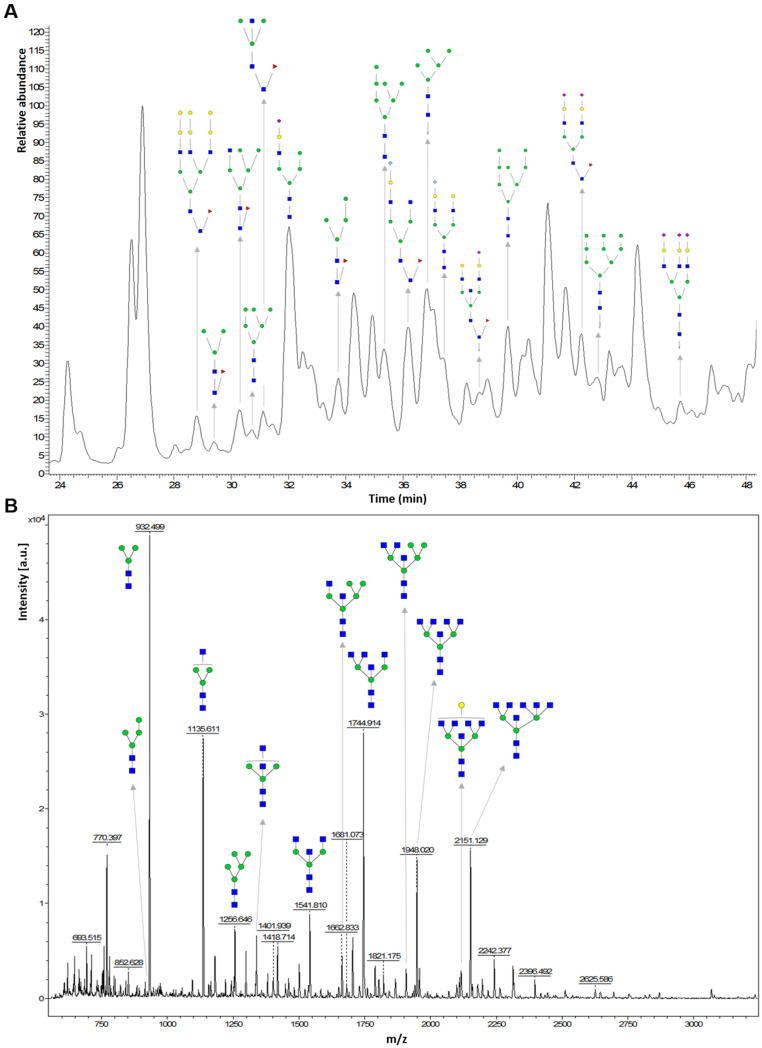
(**A**) HPLC-MS Orbitrap chromatogram of mouse brain *n*-glycans released with PNGase F and labelled with RFMS. (**B**) Positive ion MALDI-TOF spectra of *n*-glycans from ovalbumin released by treatment with PNGase F and recorded in 2,5-DHB matrix. The symbol nomenclature used for each glycan structure is shown in Figure 1.

MALDI-MS is a soft ionisation technique in which a sample is embedded in a low molecular weight ultraviolet-absorbing matrix. Ionisation is affected by a pulsed laser which will be absorbed by the matrix. This energy is then transferred to the compounds present on the sample and enables the predominant formation of singly charged quasi-molecular ions [172]. For the analysis of *n*-glycans, 2,5-diydroxybenzoic acid (DHB) is the most frequently used matrix for the suspension and co-crystallisation of the oligosaccharides, permitting their efficient ionisation. Other matrices, such as oxidised carbon nanotubes with short and open-end structures produce strong signals from small carbohydrates and amino acids [173]. Both of these matrices generally generate [M + Na]+ ions [174]. Many other matrices have been tested for the analysis of carbohydrates and glycoconjugates using MALDI-MS [174]. Figure 2B shows a characteristic MALDI-TOF MS spectra of *n*-glycans.

The use of MALDI offers several advantages compared with ESI. It has a higher salt tolerance to contaminants (for instance, salts in buffers) [164] and little susceptibility to suppression effects. However, it also has a few disadvantages, like the variations between spectra performance at different positions in the sample spots, which are often observed. In order to overcome this drawback, a spot has to be sampled in several locations until the best signal to noise ratio is obtained [175]. In addition, MALDI-MS cannot resolve isobaric compounds unless MS/MS is employed [163] and the presence of abundant ions derived from the matrix often limits the utility of MALDI-MS for the analysis of small molecules [174]. Although positive-ion MALDI-MS is predominantly used to profile glycans, negative-ion MALDI-MS has recently gained interest as it can provide advantageous fragmentation. Its main difficulty is the formation of the deprotonated ion [M-H]-, which is very successful for ESI but not for MALDI. The formation of [M+Cl]- has been proved to be a useful alternative as it produces similar fragmentation for sequencing neutral oligosaccharides using MALDI-MS/MS [176].

MALDI can also be used as a mass spectrometry imaging technique (MALDI-IMS), a well-developed tool that enables high throughput reporting of molecules localised within a single formalin-fixed, paraffin embedded (FFPE) tissue section. By using the enzyme PNGase F to release the *n*-glycans from thin tissue sections, MALDI-IMS is routinely used to examine the distribution of *n*-glycosylation [177,178].

##### Liquid Chromatography Coupled to Mass Spectrometry

MS is commonly coupled to separation techniques, mainly LC, in order to significantly improve the identification and quantitation of low abundant glycans by reducing competitive ionisation and detector saturation [179]. In addition, LC-MS is sometimes further combined with FLR for the detection of glycans for their extended dynamic range and more comparable response between released glycans. Thus, MS provides poorer quantification but is slightly more sensitive [145] compared to FLR. In positive ESI-MS there is the tendency of forming adducts with protons, sodium, potassium and ammonium, which splits the glycan signal into multiple peaks and makes quantification less robust [180]. Nevertheless, ESI-MS is optimal for the measurement of the ratio of different glycans which coelute in the same peak. Therefore, FLR and MS are two complementary detection techniques [145]. Figure 2A shows a typical LC-MS chromatogram of *n*-glycans.

As well as MALDI-MS, ESI-MS is a soft ionisation technique commonly applied to obtain a glycosylation profile. This technique consists in the nebulisation of a sample solution into electrically charged droplets, the liberation of ions from droplets, followed by the transportation of ions from the atmospheric pressure ionisation source region into the vacuum and mass analyser of the mass spectrometer [181]. ESI has a few drawbacks compared to other ionisation techniques. Its ionisation efficiency decreases when the molecular weight increases, unlike MALDI, which maintains a constant ionisation efficiency when the size of the molecule increases. Furthermore, ESI generates multiply charged ions which complicates spectral analysis. Nevertheless, the instrument software can perform a deconvolution of the multiple charge states to produce a spectrum that shows a single molecular weight distribution. This approach will solve the issue but will affect the ability to detect low-abundance species as the signal will be distributed over multiple charge states [129].

Amongst the different types of LC that can be used in the determination of glycans, nanoscale reversed-phase columns are also used [7,16,23,80,87]. These columns typically have a dimension of 75 μm × 150 mm and a flow rate of around 300 nL/min. The reduction of the internal diameter of the column limits the amount of the sample that can be injected, which is a drawback. In order to inject larger volumes, trapping columns are used [16]. The reduction of the internal diameter increases the sensitivity of the measurements with MS detection, achieving sensitivities in the low femtomole range [18]. Coupling nanoscale columns with MS detection is optimal for glycan analysis as the samples are often complex and full chromatographic separation is not obtained. In order to further improve the chromatographic separation, two-dimensional LC can be used, which is characteristic for successfully achieving the separation of isomers [182,183]. In the first dimension, separation is performed in HILIC mode at analytical scale, resulting in an incomplete separation of the different glycans in the samples. Using nanoscale reversed phase in the second dimension allows the separation of complex samples and isomer mixtures. Altogether, a two-dimensional separation is achieved obtaining detailed characterisation of complex glycan mixtures [16,182,183].

As previously mentioned, the derivatisation of glycans not only enables the optical detection of glycans but also enhances the ionisation and fragmentation behaviour in MS [18]. The aforementioned tags show differences between them. For instance, even though procainamide and 2-AB have the same mechanism, procainamide shows increased FLR and ionisation over 2-AB, as it contains a basic tertiary amine tail with high proton affinity, exhibiting a higher sensitivity in positive mode ESI-MS [184].

##### MS Fragmentation Methods

Even though glycans can be identified using high resolution MS (HRMS), the lack of characteristic fragment ions can be a problem for detailed structural elucidation of glycans. This can be solved by making molecular ions more energetic by inducing fragmentation, which can be achieved by employing tandem MS [129,152]. Among the existing fragmentation techniques, collision-induced dissociation (CID) is employed for the generation of tandem mass spectra of oligosaccharides [185], in which analyte molecules are accelerated and collided with a neutral gas, resulting in the fragmentation of molecular bonds and the creation of tandem mass spectra [186]. Dissociation of protonated glycan or glycopeptides ions is not recommended because monosaccharide rearrangements may result. On the contrary, the use of sodium (or other metal) cationised ions is more recommended since rearrangements of this kind of ions have not been reported [171].

**Figure 3:**
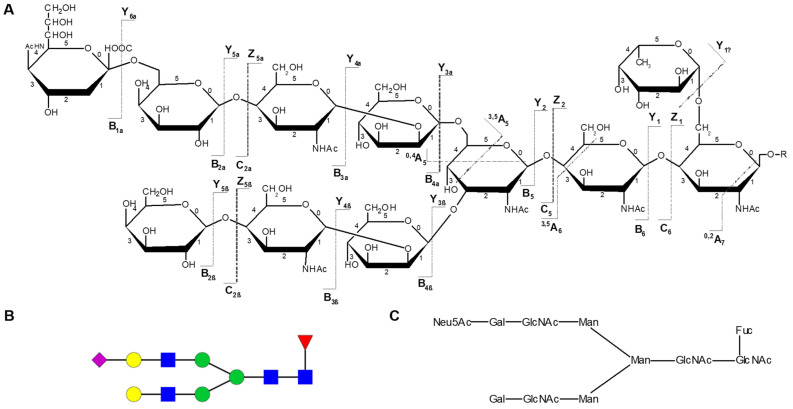
Three types of structural representations for one specific glycan. (**A**) Diagram with some of the main cleavage sites ivolved in fragment ion formation. (**B**) Symbolic representation of the glycan structure. (**C**) Tree abstraction of the glycan structure.

Generally, glycans undergo two types of cleavage upon CID: glycosidic cleavages (for instance, B-/Y- and C-/Z-types) result from the rupture between the two neighbouring residues which provide information on the composition and sequence, whereas cross-ring cleavages (A-/X- types) provide linkage information due to the fragmentation of a sugar ring [129,176]. This type of fragmentation is shown in both positive and negative ion modes, hence a common nomenclature is used, as illustrated in Figure 3. When the charge is retained on the carbohydrate portion, fragments are designated as Ai, Bi and Ci, where i represents the number of the glycosidic bond cleaved, counted from the non-reducing end (except for branched oligosaccharides, which follow a different rule). Differently, those ions containing the reducing sugar unit are labelled as Xj, Yj and Zj, where j corresponds to the number of the interglycosidic bond counted from the reducing end. The glycosidic bond linking to the reducing end is numbered 0 [187]. Focusing on the sugar ring fragmentation, in which carbon-carbon bonds are cleaved, the cross-ring cleavages are designated with Aj and Xj labels. Two superscripts, k and l (i.e., k,lAi and k,lXj) are used to indicate the sugar ring bonds that have been broken. Each ring bond is encoded with a number, as shown in Figure 3 [129].

As previously mentioned, branched oligosaccharides do not have the same nomenclature. Their carbohydrate portion is known as the “core” unit and the branches as “antennae”. Each antenna is represented by a Greek letter (α, β and γ), which are assigned with decreasing molecular (fragment) weight (α ≥ β ≥ γ). This nomenclature is then applied to branched carbohydrates [187]. When fragmentations take place on the α- and β-antennae, they are represented as Aiα, Biα, Ciα, Xjα and Zjα, respectively. Ions resulting from the core unit are designated without a Greek letter, but the numbering begun in the core continues into the antennae, and vice versa [187].

On the other hand, higher-energy collision dissociation (HCD), introduced following the development of the Orbitrap MS, is a CID related fragmentation technique in which beam-type fragmentation spectra are created inside the HCD cell. This technique can be used for the detection of individual monosaccharide fragments as it does not have the “1/3 rule” which establishes a lower range *m*/*z* cut-off which is what limits ion-trap CID [188]. 

Some of the existing automated analysis tools and software used to determine glycan composition take into account the fragmentation patterns of glycans that have just been stated above.

##### Sialic Acid Containing Glycan Analysis

Sialic acids are a family of 9-carbon containing acidic carbohydrates, which frequently terminate the glycan structure. Sialylation affects the half-lives of many circulating glycoproteins and plays important roles in several biologic processes such as cell-cell communication, cell-matrix interaction, adhesion and protein targeting [189]. This modification occurs in approximately 20% of core-fucosylated biantennary Fc *n*-glycans [43]. Moreover, these carbohydrates are found on both *n*- and O- linked glycans attached to either galactose or *n*-acetylglucosamine via α2,3- or α2,6-linkages whose syntheses are catalysed by specific enzymes [78].

The presence of sialic acids can complicate the quantification of both neutral and acidic glycans in a single sample, as neutral glycans ionise better in positive mode whereas sialylated glycans ionise better in negative mode. Sialic acid residues are highly labile on sialylated carbohydrates, hence, in source fragmentation with both MALDI and ESI can lead to sialic acid residue loss, which consequently complicates structural and quantitative analysis of these glycans [190]. To overcome this problem, several derivatisation strategies can be applied in order to neutralise sialic acid residues. These strategies allow the simultaneous analysis of neutral and acidic glycans apart from preventing the loss of the sialic acid residue due to in-source fragmentation [164].

Permethylation is the predominant derivatisation technique applied to sialylated glycans. The ionisation efficiency of glycans is improved when they become less hydrophilic by converting their polar hydroxyl and carboxylate groups into nonpolar methylated homogenous derivatives [164,191]. This derivatisation method offers several advantages regarding the analysis of glycans as it helps facilitate the determination of sequence and composition of monosaccharides in glycans, branching position and interglycosidic linkage information, as well as the presence of configurational and conformational isomers [192]. Permethylation enhances the stabilisation of sialic acid residues through neutralisation, enabling the simultaneous analysis of neutral and acidic carbohydrates in positive ion mode [164]. Moreover, this technique facilitates structure elucidation by tandem MS, through the formation of more informative fragments. However, the isomeric separation of permethylated glycans using the existing methods is not satisfactory [188].

Esterification is an alternative to permethylation, which was originally achieved by the formation of ethyl esters using methyl iodide in DMSO [193]. More recently, 3-methyl-1-p-tolytriazene (MTT) in DMSO-acetonitrile solution [194] or 4-4(4,6-dimethoxy-1,3,5-triazin-2-yl)-4-methyl-morpholinium chloride (DMTMM) in methanol have been used. This last derivatisation method offers the advantage that α2,3- and α2,6- linked sialic acids can be differentiated, as methyl esters are produced from α2,6-linked acids and lactones from α2,3-linked acids. This fact allows linkage position to be determined by the resulting mass difference, avoiding the use of exoglycosidase digestions for the linkage determination [164,195]. 1-ethyl-3-(3-(dimethylamino) propyl) carbodiimide (EDC) and 1-hydroxybenzotriazole (HOBt) in an ethanolic solution are also employed as an esterification method. These reagents are widely used in peptide synthesis, in which EDC is responsible for initial carboxylic acid activation and HOBt catalyses the subsequent conversion to an ester or an amide [196].

Finally, amidation is another derivatisation strategy in which several reagents can be employed. Complete amidation of both α2,3- and α2,6- sialic acids can be achieved by using acetohydrazide and (EDC) as a coupling reagent in mild acidic conditions. Azetohydrazide can be easily coupled with the carboxylic acid; however, the aldehyde of the reducing end of a free glycan is also reactive to hydrazide. Therefore, permanent charge cannot be incorporated after the amidation reaction [197]. To overcome this issue, amidation can be performed on the sialylated *n*-glycan while it is still attached to the protein [198]. EDC, HOBt and dimethylamine in DMSO are also employed in amidation techniques [199], as well as DMT-MM in ammonium chloride [200].

##### Isotopic Labelling

Isotopic labels are widely used for quantitative analysis. They offer a major advantage for glycan analysis as they enable the simultaneous analysis of multiple samples and comparison with an internal standard. The use of these labels provide a method for the separation of derivatised and underivatised glycans [201]. Internal standards with similar characteristics to the analytes have also been used to standardise analysis to a known quantity or to perform absolute quantitation [163,202]. However, this practice is not widely used as the availability of internal standards is limited and molar differences in glycan ionisation introduce large errors when standards of similar characteristics are used [163,164,203].

Chemical or metabolic labelling approaches incorporate a heavy isotope into a glycan for comparative analysis. These techniques help reduce some issues related to label free analysis such as instrument response and differences in ionisation efficiencies. In chemical labelling approaches, heavy isotopes are introduced during methylation or reductive amination procedures for the analysis of glycans using MALDI-MS [204] or LC-ESI-MS [205].

For instance, the reagents 13CH3I and 12CH2DI were used during methylation to analyse *n*-linked oligosaccharides from glycoprotein and human serum [206]. The mass difference between these compounds is only 0.002922 Da which required high resolution MS with more than m/Δm = 30,000 resolution in order to resolve the two isotopic species. This method is known as QUIBL, used for quantitative isobaric labelling and particularly useful for the quantitation of isomers by MS/MS [202]. Isotopic labelling is also a suitable approach for those glycans that cannot be modified by reductive amination. This is the case of O-glycans that are released through β-elimination, producing a hydroxyl group at the reducing end which impedes the use of reductive amination. In order to overcome this issue, isotopic labelling using NaBD4 during β-elimination can introduce deuterium labels [207].

##### Ion Mobility

In recent years, IM has been used in the field of glycomics for the separation of isomeric compounds. In this technique, ions are not only separated according to their mass and charge but also according to their shape and size. Hence, ion-mobility mass spectrometry (IMMS) can be an optimal technique for the separation of glycoconjugate isomers which have different sialic acid linkage [208,209]. When IM is coupled on-line with MS, it provides three-dimensional analytical information for each detected species: shape-to-charge, mass-to-charge and abundance, allowing reliable analyte identification [208,209].

Specifically, IM measures the time (drift time) that a particular ion takes to cross a cell filled with an inert, neutral background gas (commonly N2 or He) at a controlled pressure under the influence of a weak electric field. The drift time of a specific ion is mainly due to ion-gas collisions, hence ions are separated due to their ion-neutral collision cross-section (Ω), related to the overall shape and topology of the ion [209,210]. Furthermore, the accelerating electric force is greater when the charge of the ion is higher, meaning that the ion will cross the chamber more quickly. Taking this into account, the drift time of an ion is determined by the collision cross-section-to-charge ratio (Ω/z) [211]. In order to further expand the utility of IMMS to glycomics, separation factors will need to be augmented with multiple stages of IMMS or orthogonal analysis techniques such as gas-phase action spectroscopy [208].

##### MS Analysis Tools

There are many analysis tools used for the identification of glycans and glycopeptides from both MS and MS/MS data, as reported by Woodin et al. [3]. GlycoWorkbench is one of the most commonly used to identify both glycans and glycopeptides from MS data. It looks for matches between calculated theoretical glycan masses and the corresponding *m*/*z* values from a spectral peak list uploaded by the user [212]. Similarly, GlycoSpectrumScan identifies *n*- and O-linked glycoforms using MS data, as well as determining the relative abundance of these glycoforms for each glycosylation site [3,213]. Many other tools are also used, such as Glycofragment and GlycoSearchMS, both developed for glycan structure determination. Glycofragment calculates the theoretical fragmentation patterns of glycan structures and GlycoSearchMS compares the experimental data obtained with the theoretical spectra from *n*-linked and O-linked glycan fragmentation entries extracted from SweetDB [214].

SimGlycan is an ideal tool to increase throughput of glycan analysis. Glycan structures can be determined from MS/MS data obtained from various mass spectrometers, using a built-in database with theoretical fragmentation profiles to provide the most likely structure candidates. SimGlycan stands out from other software platforms for its capacity to report novel glycans. Glycan structures are determined, monosaccharide by monosaccharide, from the fragments observed. Furthermore, the software has been updated to perform fragmentation analysis for glycopeptides [207]. ProteinScape is another software used for the identification of glycans and glycopeptides [80,215], amongst many others that are either freely available or not [3].

In addition to the available analysis tools and software for the identification of glycans, several glycomic databases are available, which document different glycan structures. As reviewed by Hizal et al. [216], the Consortium Functional Glycomics (CFG) glycan structure database [217], Glycobase [212], Glycome DB [218], GlycoSuiteDB [219], EuroCarbDB [220] and Lectin Frontier Database [221] are a few of the publicly accessible databases.

#### 3.2.3. Nuclear Magnetic Resonance

NMR is a very powerful tool for the analysis of complex *n*-glycans and has specifically been proven to be suitable for the determination of the primary sequence of glycans [27]. NMR is a nondestructive technique as it leaves the sample intact for further analyses and, with sample amounts as small as 15 pmol [215]. This technique can provide structural information for isolated glycan species, however, the quantities required to achieve actionable levels of signal to noise ratio in NMR are a significant challenge for those studies targeting low abundance glycans [208]. However, more recent studies have demonstrated that NMR can indeed be highly sensitive if water suppression and sample preparation are optimised [215].

In order to interpret the 1H-NMR spectrum of a carbohydrate chain in terms of primary structural assignments, Vliegenthart and Kamerling developed the structural reporter group (SRG) concept. The SRG concept is based on the fact that the chemical shifts of specific glycan protons are very sensitive to the structure of a given glycan, and the comparison of structural elements allow the characterisation of the compound [222].

Therefore, NMR data offers additional information which can be tightly integrated with LC-MS and MS/MS data, achieving a complete characterisation of even isobaric glycans differing in only one linkage position or in the substitution in one branch [215].

## 4. Conclusions

Glycome analysis is emerging as a source of potential biomarkers in different pathological states [223]. Its analysis is not easy, as the diverse structures of glycans are complex and heterogenic. Moreover, there is a wide range of possible monosaccharide combinations and linkages, that result in structurally complex glycans, which can be attached to proteins, conforming glycosylation PTMs.

A wide variety of glycans have been reported to be altered in many studies focused on determining the glycosylation profile in ageing and several age-related diseases. Galactosylation and sialylation are altered in many diseases; however, these features specifically decrease with age, as well as in AD and RA patients, but increase in ALS patients. Fucosylation is increased in several types of cancer and decrease in IBD and T2DM patients. The glycan patterns shown amongst the different types of age-related diseases are limited, nevertheless, aberrant glycosylation could help distinguish different diseases and identify potential diagnostic and prognostic biomarkers.

In clinical studies, the comparison of glycans levels altered in specific diseases often leads to inconsistent results, which cannot be explained completely by the different statistical methods. This may be due to the diversity of the glycome in different populations and even in different environments. Diseases can show diversity among populations, justifying the inconsistency of some results. However, glycan traits can be considered as a new avenue of research in the systematic understanding of complex diseases.

Furthermore, the use of a wide range of glycomic methodologies leads to low comparability between studies which hinders the obtention of clear results. CE is characteristic for providing fast and efficient separations, as well as being highly automated, whereas IMMS is considered to be optimal for the separation of glycoconjugate isomers which have different sialic acid linkage. NMR is a nondestructive technique as it leaves the sample intact for further analyses and can provide structural information for isolated glycan species. Nevertheless, MS seems to be the most promising technique, with the possibility to use different types of chromatographic separation combined with the most appropriate ionisation technique and mass analyser.

## Figures and Tables

**Table 1:** Glycan alterations on chronological ageing.

Sample Type	Glycan Alteration	Technique	Sample Treatment	Citation
Serum	Increase in under-galactosylated glycans and decrease in a core α-1,6-fucosylated bigalactosylated biantennary structure in individuals with more than 40–50 years of age.	DSA-FACE	Purification of immunoglobulins with protein L, denaturation, *n*-glycan release with PNGase F, sialidase treatment and APTS labelling.	[22]
Plasma	Increase of non-galactosylated glycans (A2 and FA2) and decrease of digalactosylated glycans (A2G2, FA2G2, A2BG2 and FA2BG2). Monogalactosylated glycans increase or decrease depending on the position of the galactose and the presence of bisecting GlcNAc.	HILIC-FLR	IgG isolation using protein G monolithic plates, “in gel” *n*-glycan release with PNGase F and 2-AB labelling.	[48]
Plasma	Non-galactosylated (A2, FA2 and FA2B) and monogalactosylated (FA2(6)BG1 and FA2(3)BG1) glycans steadily increase with age, compared to digalactosylated glycans (A2BG2, FA2G2, FA2G2S1 and FA2BG2S2) which decrease.	HPLC-FLR/MS	IgG isolation using protein G monolithic plates, denaturation, “in solution” *n*-glycan release, 2-AB labelling and HILIC-SPE purification.	[49]
Serum	Increased α2,6 sialic acid, mannose, *n*-acetylglucosamine and multiantennary complex type *n*-glycans.	Lectin-Based Protein Microarray	Isolation of α2M using a Co-Immunoprecipitation Kit, incubation with 8 different lectins and labelling with CF647-streptavidin conjugate.	[50]
Serum	The log of the ratio of two glycans (NGA2F and NA2F), named GlycoAgeTest, remains steady up to the age of 40 years and thereafter gradually increases. Patients with dementia or Cockayne syndrome have a higher GlycoAgeTest level than age-matched healthy individuals.	DSA-FACE	Denaturation, *n*-glycan release with PNGase F, APTS labelling and desialylation.	[11]
Plasma	Significant differences in glycan microheterogeneity, with an increased sialic acid content released from newborn umbilical cord (NUCP) α-2-macroglobulin (A2M).	FACE	Neuraminidase digestion, and use of electrophoresis, Western blotting and immunostaining to determine the degree of sialylation and terminal galactosylation. *n*-glycan profile: A2M purification with immunoprecipitation, denaturation, *n*-glycan release with PNGase F, protein precipitation, evaporation and 7-amino-1,3-naphthalenedisulfonic acid (ANDS) labelling.	[51]
Placenta	Differences in the abundance of high mannose *n*-glycans, 2- sialylated biantennary *n*-glycans, 3- (core)fucosylated biantennary *n*-glycans, 4- highly fucosylated *n*-glycans, 5- bisected *n*-glycans and 6- multiantennary *n*-glycans.	MALDI-TOF MS	Denaturation, reduction, alkylation, *n*-glycan release with PNGase F, glycan purification with porous graphic carbon columns, permethylation for the stabilisation of sialic acids, further purification with C18 columns and mixture with 2,5-DHB matrix.	[52]
Serum and pituitary extracts	Most TSH molecules are low-*n*-glycosylated, highsulfonated and low-sialylated in children up to 18 months, compared with older children and adults. The degree of *n*-glycosylation is similar in serum and pituitary extracts up to 3 months of age and after that is higher in serum than in pituitary extracts.	Competitive binding radioimmunoassay and noncompetitive time-resolved sandwich fluoroimmunoassay	Use of electrophoresis to measure frequencies of glycoforms and neuraminidase treatment to determine sialic acids in serum and pituitary extracts. Homogenisation of pituitary extracts and quantification of TSH with the two immunoassays.	[53]
Skin	Significant quantitative decreases in high mannose glycans in aged skin.	HILIC-FLR	-	[54]
Plasma	Galactosylation and sialylation decrease with increasing age and show significant sex dependence. Females in their 45 to 60 years show the most prominent drop in the levels of galactosylated and sialylated glycoforms. The incidence of bisecting *n*-acetylglucosamine increases in younger individuals and reaches a plateau at older age.	MALDI-TOF-MS and HILIC-FLR	IgG isolation using protein G monolithic plates. *n*-glycan release with PNGase F and 2-AB labelling.	[55]
Plasma	Galactosylation tends to decrease with age as di-galactosylated glycopeptides are less abundant in older participants, while nongalactosylated glycopeptides are more abundant in older participants.	MALDI-TOF-MS	Glycopeptides: IgG isolation using a protein A affinity purification step, trypsin digestion and purification with C18-SPE plate and mixture with a-cyano-4-hydroxy-cinnamic acid matrix	[56]
Plasma	Bisection, galactosylation, sialylation of diantennary species and tetraantennary species, as well as the size of high-mannose species are important plasma characteristics associated with inflammation and metabolic health.	MALDI-FTICR-MS	Protein denaturation, *n*-glycan release with PNGase F, 2-aminobenzoic acid (2-AA) labelling, HILIC-SPE purification and carbon-SPE desalting.	[57]
Plasma	Glycosylation patterns of α1-antitrypsin (αAT) enriched fractions are associated with chronological age and differ between females and males. Pronounced differences exist between males and females in the glycosylation profiles of immunoglobulin A enriched fractions.	CE-LIF	Protein denaturation, *n*-glycan release with PNGase F, APTS labelling and HILIC-SPE purification.	[58]
Serum	Age-related changes are observed in *n*-glycans NGA2F, NGA2FB and NA2F (agalactosylated core-α-1,6-fucosylated biantennary glycan, core-α-1,6-fucosylated bisecting biantennary glycan and bigalactosylated core-α-1,6-fucosylated biantennary glycan, respectively). Furthermore, fucosylation of *n*-glycans is significantly different between men and women: more core-α-1,6-fucosylated glycans are detected in women, whereas more branching-α-1,3-fucosylated *n*-glycans are seen in men.	DSA-FACE	Protein denaturation, *n*-glycan release with PNGase F, sialidase treatment (neuraminidase) and APTS labelling.	[59]

**Table 2:** Glycan alterations on neurodegenerative diseases.

Disease	Sample Type	Glycan Alteration	Technique	Sample Treatment	Citation
Alzheimer’s disease	Plasma	Lower abundance of complex galactosylated and sialylated forms in patients with AD.	NanoLC-MS/MS (Orbitrap)	Reduction, alkylation, trypsin digestion and sialidase digestion.	[24]
Amyotrophic lateral sclerosis	Serum	High levels of sialylated glycans and low levels of core fucosylated glycans in patients with ALS, compared to healthy volunteer sera.	MALDI-TOF MS, HPLC-FLR	IgG purification with protein G beads, reduction, IgG separation with SDS-PAGE, *n*-glycan release with PNGase F, *n*-glycan extraction from gel pieces, decontamination with ion-exchange resin, 2-AB labelling and further exoglycosidase digestion. Desalting and mixture with DHB for MALDI-TOF MS analysis.	[68]
Amyotrophic lateral sclerosis	Cerebrospinal fluid	Detection of diantennary *n*-glycans predominantly with proximal fucose and some bisecting GlcNAc; agalacto-, mono- and digalactosylated as well as α2,6-sialylated structures in ALS patients. Furthermore, increased levels of galactosylated structures in ALS patients.	HPLC-FLR, MALDI-TOF-MS	IgG isolation with protein G cartridge, evaporation, protein precipitation and denaturation, *n*-glycan release with PNGase F, purification with porous graphitic carbon cartridges, evaporation and 2-AB labelling. Further exoglycosidase treatment for structure elucidation. Mixture with DHB for MALDI-TOF-MS analysis.	[69]
Amyotrophic lateral sclerosis	Cerebrospinal fluid	Determination of complex diantennary structures with sialic acid in α2,3- and α2,6-linkage, bisecting *n*-acetylglucosamine-containing structures as well as peripherally 30 fucosylated structures. Increase of monosialylated diantennary glycans A2G2S(6)1 and FA2G2S(3)1 in ALS.	HPLC-FLR, RP-HPLC/MS/MS	Protein precipitation, denaturation, reduction, trypsin digestion, purification with C18 cartridges, evaporation, *n*-glycan release with PNGase F, separation and desalting of *n*-glycans with C18 and porous graphitic carbon cartridges, respectively. 2-AB labelling and purification by gel filtration. Further exoglycosidase treatment for structure elucidation.	[70]
Parkinson’s disease	Plasma	PD patients showed a reduced relative abundance of a high-mannose *n*-glycan structure, a monosialylated *n*-glycan structure and a core fucosylated monosialylated *n*-glycan structure with an additional fucose attached to one antenna, as well as an increased relative abundance of a core fucosylated monogalactosylated *n*-glycan structure.	HILIC-FLR	IgG isolation using protein G monolithic plates, evaporation, denaturation, *n*-glycan release with PNGase F, 2-AB labelling and HILIC-SPE purification.	[40]
Parkinson’s disease	Serum	Low sialylation and increased fucosylation is increased in PD patients on tri-antennary glycans with 2 and 3 terminal sialic acids.	CE-MS/MS	Denaturation, reduction, alkylation, *n*-glycan release with PNGase, filtering, evaporation, hydrolysis of the glycosylamines, 2-AA labelling and HILIC purification.	[71]

**Table 3:** Glycan alterations on cancer.

Disease	Sample Type	Glycan Alteration	Technique	Treatment	Citation
Hepatocellular carcinoma	Serum	One triantennary glycan (NA3Fb) is correlated with tumour stage in HCC patients.	DSA-FACE	Purification of immunoglobulins with protein L, denaturation, *n*-glycan release with PNGase F, sialidase treatment and APTS labelling.	[74]
Breast cancer	Serum	Increases in sialylation and fucosylation of glycan structures appear to be indicative of cancer progression. Changes in the relative intensities of 8 *n*-glycans are characteristic of breast cancer (*n*-glycans sialylated to a different degree (mono-, di-, tri- and tetrasialylated) and 5 of these structures are fucosylated (2 of them difucosylated)).	MALDI-MS	Reduction, alkylation, trypsin digestion, *n*-glycan release with PNGase F, purification with activated charcoal microcolumns, permethylation and mixture with 2,5-DHB matrix.	[75]
Breast cancer	Serum	Increase in siaylation and changes in fucosylation in breast cancer patient sera compared to that from controls. Furthermore, patients show elevated levels of the sLex-carrying triantennary structure, A3FG1, derived from the monofucosylated trisialylated triantennary *n*-glycan (A3FG3S3).	CapLC-QTOF	In-gel *n*-glycan release with PNGase F and 2AB labelling/reduction, alkylation, *n*-glycan release with PNGase F and in-gel trypsin digestion.	[76]
Breast cancer	Serum	Presence of 15 unique serum glycan markers in all patients but absent in normal individuals.	MALDI-FT-ICR	β-elimination, glycan purification with a graphitised carbon cartridge and mixture with a DHB and DHAP matrix.	[77]
Breast cancer	Serum	Increased levels of α2,3 sialylation in breast cancer samples.	MALDI-TOF-MS	Trypsin digestion, denaturation, reduction, *n*-glycan release with PNGase F, purification with C18 micro-spin columns and Graphite micro-spin columns, sialic acid amidation and clean-up and solid-phase permethylation	[78]
Breast cancer	Serum	Breast cancer patients exhibit a characteristic pattern of IgG Fc region *n*-glycosylation.	MALDI-MS	IgG isolation, SDS-PAGE, enzymatic glycan release, methylamidation of *n*-glycan sialic acid and AQ-labeling.	[26]
Gastric cancer	Serum	9 *n*-glycan structures altered and decrease of core-fucosylated structures.	DSA-FACE	Enzymatic glycan release, 8-aminonaphthalene-1,3,6-trisulfonic acid disodium salt (ANTS) labelling and sialidase digestion.	[79]
Pancreatic cancer	Serum	Aberrant glycosylation in four proteins (LIFR, CE350, VP13A, HPT) found in sera from pancreatic cancer patients compared to those of controls.	NanoLC-MS/MS	Immunodepletion, incubation with PHA-L lectin, reduction, alkylation, PNGase F and trypsin digestion.	[80]
Lung cancer	Plasma and serum	Significant elevation of α2−6 sialylation, β1−4 branching, β1−6 branching, antennary fucosylation and total *n*-glycosylation level in almost every stage of lung cancer relative to control groups.	GC-MS	Permethylation, nonreductive release, purification, hydrolysis, reduction and acetylation.	[81]
Colorectal cancer	Serum	Increased degree of fucosylation in CRC patients.	Lectin Based Protein Microarray	Transferrin isolation, incubation with fourteen biotinylated lectins, wash with PBST and exposition to CF647-streptavidin conjugate.	[82]
Colorectal cancer	Plasma	Increased levels of sialylation and fucosylation.	Lectin blot analysis	Delipidation, immunodepletion and incubation with agarose-bound lectins.	[83]
Colorectal cancer	Serum	Decreased levels of total core fucose residues.	DSA-FACE	Enzymatic glycan release, 8-Aminonaphthalene-1,3,6-trisulfonic acid disodium salt (ANTS) labelling and sialidase digestion.	[84]
Colorectal cancer	Serum	Increased α2,6Sia, GlcNAc and mannose (Man) residues, as well as increased multiantennary complex type *n*-glycans.	Lectin Based Protein Microarray	α2M isolation by immunoprecipitation, incubation with set of lectins and streptavidin labelling.	[85]
Prostate cancer	Serum	Four high-mannose (Man6-Man9) type, one neutral and one acidic complex-type glycans are down-regulated in the patient group while one acidic complex-type glycan is up-regulated in the patient group with active disease.	MALDI-FT-ICR	Denaturation, enzymatic release, ethanol precipitation and SPE purification.	[86]
Ovarian cancer	Plasma	Up-regulated fucosylated glycans in healthy samples when compared to cancerous and benign tumour control samples.	MALDI-FT-ICR MS and NanoLC-MS/MS (Orbitrap)	Dialysis, b-elimination, SPE with graphitised carbon cartridges and pronase digestion.	[87]
Cancer	Serum	Significantly increased monofucosylated *n*-glycans at all glycosylation sites in all cancer samples. Increased core-type fucosylated *n*-glycans in gastroenterological cancer samples, increased core-type fucosylated *n*-glycan in prostate cancer samples and increased Lewis-type fucosylated *n*-glycan in metastatic prostate cancer and gastroenterological cancer.	HPLC-MS	Haptoglobin purification, reduction, alkylation, lysylendopeptidase, trypsin and endoprotease Glu-C digestion, affinity separation with Sepharose CL4B and desialylation.	[88]

**Table 4:** Glycan alterations on T2DM.

Sample Type	Glycan Alteration	Technique	Treatment	Citation
Plasma	Decreased agaloctosylated glycans without galactose and increased monogalactosylated glycans and fucosylated structures with bisecting GlcNAc.	HPLC-FLR	IgG isolation using protein G monolithic plates, “in solution” and “in gel” glycan release and labelling using PNGase F and 2-aminobenzamide (2-AB), respectively.	[8]
Plasma	Decreased galactosylation and sialyation, increase in fucosylated structures with bisecting GlcNAc and decrease in fucosylated structures without bisecting GlcNAc.	HPLC-FLR	IgG isolation using protein G monolithic plates, denaturation, glycan release, 2-AB labelling and HILIC-SPE purification.	[9]
Serum	Reduced α(1,6)-linked arm monogalactosylated and core-fucosylated diantennary *n*-glycans (NG1[6]A2F).	DSA-FACE	Denaturation, *n*-glycan release with PNGase F, APTS labelling and sialidase digestion.	[19]
Plasma	Decreased galactosylated glycan structures and increased agalactosylated glycan structures.	HPLC-FLR	IgG isolation using protein G monolithic plates, denaturation, “in solution” glycan release, 2-AB labelling and HILIC-SPE purification.	[25]
Plasma	Compared to controls, T2DM patients show decreased core fucosylated glycans, decreased levels of low-branching and increased levels of high branching plasma *n*-glycans, as well as statistically significantly increased levels of di (S2) and trisialylated (S3) plasma *n*-glycans.	HILIC-FLR	Denaturation, *n*-glycan release with PNGase F, 2-AB labelling and HILIC-SPE purification.	[91]
Plasma	Eighteen glycosylation features are significantly associated with T2DM. Fucosylation and bisection of diantennary glycans are decreased in diabetes, α2,6-linked sialylation is increased and α2,3-linked sialylation of triantennary glycans is decreased.	MALDI-TOF MS	Denaturation, *n*-glycan release with PNGase F, ethyl-esterification of sialic acids, purification with GHP membrane plate and mixture with super-DHB matrix.	[92]
Plasma	GlycA, a glycoprotein biomarker, is associated with incident T2DM.	NMR	Separation of proteins from lipoproteins with the addition of sodium bromide and further centrifugation and filtering with 10 kDa filters.	[93]

**Table 5:** Glycan alterations on metabolic syndrome and related diseases.

Disease	Sample Type	Glycan Alteration	Technique	Treatment	Citation
Metabolic Syndrome	Plasma	Specific *n*-glycan structural features (trigalactosylated, biantennary, triantennar, core-fucosylate, monosialylated, disialylated and trisialylated glycans) are significantly correlated with MetS related risk factors.	HILIC-FLR	Reduction, *n*-glycan release with PNGase F, 2AB labelling and sialydase digestion.	[95]
Metabolic Syndrome	Serum	Significantly elevated levels of NGA2FB and NA3F and lower level of the α(1,6)-arm monogalactosylated glycan (NG1A2F) in women with MetS.	DSA-FACE	Denaturation, *n*-glycan release with PNGase F, APTS labelling and sialidase digestion.	[20]
Hypertension	Plasma	Decrease of galactosylation in IgG subclasses IgG1, IgG2/3 and IgG4 with increasing blood pressure.	NanoHPLC-MS	IgG isolation using protein G monolithic plates, trypsin digestion, reverse-phase desalting and purification.	[96]
Hypertension	Plasma	Five glycans (IgG with digalactosylated glycans) significantly differ in participants with prehypertension or hypertension compared to those with normal blood pressure, while 17 other glycan traits significantly differ in participants with hypertension compared to those of normal blood pressure.	HILIC-FLR	“In solution” denaturation, “in gel” enzymatic glycan release with PNGase F and 2-aminobenzamide labelling.	[97]
Hypertension	Plasma	Ten IgG *n*-glycan traits (i.e., IgG1G0F, IgG2G0F, IgG2G1FN, IgG2G1FS, IgG2G2S, IgG4G0F, IgG4G1FS, IgG4G1S, IgG4G2FS and IgG4G2N) representing galactosylation and sialylation are significantly associated with hypertension.	NanoRP-HPLC-MS	IgG isolation by affinity chromatography and trypsin digestion.	[98]
Cardiometabolic disease	Plasma	Two agalactosylated glycans and a glycan containing a bisecting GlcNAc are significantly higher in participants with MetS compared to controls, whereas a higher level of a digalactosylated *n*-glycan is present in participants without MetS.	HILIC-MS	IgG isolation using protein G monolithic plates, denaturation, “in solution” *n*-glycan release, 2-AB labelling and HILIC-SPE purification.	[21]
Atherosclerotic cardiovascular disease	Serum	A large number of *n*-glycan traits related to core-fucose and bisecting GlcNAc are strongly associated with atherosclerotic plaque. One specific trait related to the sialylated *n*-glycan appears to be strongly negatively related to circulating VLDL and is supportive of a role of IgG glycosylation in VLDL metabolism and arterial lesion formation also in humans.	HILIC-FLR	IgG isolation using protein G monolithic plates, denaturation, *n*-glycan release with PNGase F, 2-AB labelling and HILIC-SPE purification.	[99]
Chronic kidney disease	Plasma	Altered glycans with galactosylation, sialylation and bisecting *n*-acetylglucosamine features.	HPLC-FLR	IgG isolation using protein G monolithic plates, denaturation, glycan release, 2-AB labelling and HILIC-SPE purification.	[100]
Dyslipidaemia	Plasma	Possible association between blood lipids and the loss of galactose and sialic acid. Moreover, the addition of bisecting GlcNAcs might be related to the chronic inflammation accompanied with the development and procession of dyslipidaemia.	HILIC–UPLC	IgG isolation using protein G monolithic plates, “in solution” *n*-glycan release with PNGase F and 2-AB labelling.	[101]

**Table 6:** Glycan alterations on Chronic inflammatory diseases.

Disease	Sample Type	Glycan alteration	Technique	Treatment	Citation
Inflammatory bowel disease	Plasma	Lower levels of IgG galactosylation compared to controls.	nanoLC-MS	IgG purification by Protein G affinity chromatography and tryptic digestion.	[23]
Inflammatory bowel disease	Serum	Decreased IgG galactosylation and proportion of sialylated structures.	HILIC-UPLC	IgG isolation using protein G monolithic plates, denaturation, “in solution” denaturation, *n*-glycan release with PNGase F, 2-AB labelling and HILIC-SPE purification.	[110]
Inflammatory Bowel Diseases	Plasma	Higher abundance of large-size glycans in IBD patients compared with controls, a decreased relative abundance of hybrid and high-mannose structures, lower fucosylation, lower galactosylation and higher sialylation (α2,3- and α2,6-linked).	MALDI-TOF-MS	Denaturation, *n*-glycan release with PNGase F, esterification of sialic acids, HILIC purification with a GHP membrane and mixture with super-DHB matrix or DHB matrix.	[112]
Inflammatory Bowel Disease	Serum	The agalactosyl fraction of the fucosylated IgG oligosaccharides is significantly greater in IBD patients compared to healthy volunteers. The extent of agalactosylation of IgG correlates with disease activity of IBD and is a potentially effective diagnostic marker for IBD.	RP HPLC-FLR	IgG purification using protein G sepharose, *n*-glycan release with PNGase F and 2-aminopyridine labelling.	[115]
Rheumatoid arthritis	Serum	Treatment with methotrexate or/and Remicade indicates an increase of IgG galactosylation.	Modified ELISA-plate test, biosensor BIAcore, GC-MS	Isolation of IgG by affinity chromatography on Protein A-Sepharose column, protein denaturation, reduction and dialysis. Hydrolysation, evaporation and neutralisation for GC-MS analysis. Elisa-plate test: Reduction of purified IgG, interaction with two lectins and ExtrAvidin-AP conjugation. Biosensor BIAcore: Lectin immobilisation and measurement of the binding.	[116]
Rheumatoid arthritis	Serum	Peaks of glycans with agalactosylated glycan structures are increased in Rheumatoid arthritis cases.	HILIC -FLR	IgG isolation using protein G monolithic plates, denaturation, *n*-glycan release with PNGase F, 2-AB labelling and HILIC-SPE purification.	[117]
Rheumatoid arthritis	Serum	Statistically significant increases in bisecting glycans FA2BG2 and FABG2S1 seropositive RA, accompanied by decrease of bisecting monogalactosylated glycan FA2[6]G1 and non-bisecting monosialylated glycan FA2[3]G1S1.	CE-LIF	Isolate IgG with protein A microwell plate, *n*-glycan release with PNGase F, APTS labelling and clean-up.	[118]
Rheumatoid arthritis	Serum	Aberrant galactosylation of IgG in RA compared to healthy controls. Significant correlation between levels of aberrant IgG galactosylation and disease activity (higher in females than males).	HPLC-FLR	Purify IgG using a protein G HP column, reduce, alkylate and immobilise in SDS-polyacrylamide gel matrix. Release glycans with PNGase F and label with 2-aminobenzamide	[119]
Rheumatoid arthritis	Serum	Patients with RA have a decrease in galactose content in IgG, which is associated with the disease activity, disease duration and stage of joint destruction.	GC	Purification of IgG, evaluation of neutral monosaccharides through phenolsulfuric acid method, methanolysis and silylation.	[120]
Rheumatoid Arthritis	Plasma	Structure GP1 (agalactosylated glycan) might have potential as a putative biomarker for RA in the Han Chinese population, while the change in IgG glycosylation shows association with the RA active and remission states.	HPLC-FLR	IgG isolation with protein G plate, reduction, alkylation, in-gel *n*-glycan release with PNGase F, 2-AB labelling, SPE purification.	[121,122]
Idiopathic inflammatory myopathies	Serum	IIM patients contain less galactosylated epitopes compared to healthy controls and the Fc-glycan profile of Jo1+ patients contains less bisected and afucosylated glycans compared to Jo1− patients.	nanoRP-LC-MS/MS (Orbitrap)	Glycopeptides: IgG isolation, reduction, alkylation, trypsin digestion, desalting with C18 plates and evaporation.	[123]

## Data Availability

Not applicable.

## References

[1] Van Den Steen P., Rudd P.M., Dwek R.A., Opdenakker G. (1998). Concepts and principles of O-linked glycosylation. Crit. Rev. Biochem. Mol. Biol..

[2] Miura Y., Endo T. (2016). Glycomics and glycoproteomics focused on aging and age-related diseases—Glycans as a potential biomarker for physiological alterations. Biochim. Biophys. Acta Gen. Subj..

[3] Woodin C.L., Maxon M., Desaire H. (2014). Software for automated interpretation of mass spectrometry data from glycans and glycopeptides. Analyst.

[4] Ohtsubo K., Marth J.D. (2006). Glycosylation in cellular mechanisms of health and disease. Cell.

[5] Dennis J.W., Granovsky M., Warren C.E. (1999). Protein glycosylation in development and disease. BioEssays.

[6] Handerson T., Camp R., Harigopal M., Rimm D., Pawelek J. (2005). B1,6-branched oligosaccharides are increased in lymph node metastases and predict poor outcome in breast carcinoma. Clin. Cancer Res..

[7] Anugraham M., Jacob F., Nixdorf S., Everest-dass A.V., Heinzelmann-schwarz V., Packer N.H. (2014). Specific glycosylation of membrane proteins in epithelial ovarian cancer cell lines: Glycan structures reflect gene expression and DNA methylation status. Mol. Cell Proteomics.

[8] Li X., Wang H., Russell A., Cao W., Wang X., Ge S., Zheng Y., Guo Z., Hou H., Song M. (2019). Type 2 diabetes mellitus is associated with the immunoglobulin G *n*-glycome through putative proinflammatory mechanisms in an Australian population. OMICS.

[9] Lemmers R.F.H., Vilaj M., Urda D., Agakov F., Šimurina M., Klaric L., Rudan I., Campbell H., Hayward C., Wilson J.F. (2017). IgG glycan patterns are associated with type 2 diabetes in independent European populations. Biochim. Biophys. Acta Gen. Subj..

[10] Kailemia M.J., Park D., Lebrilla C.B. (2017). Glycans and glycoproteins as specific biomarkers for cancer. Anal. Bioanal. Chem..

[11] Vanhooren V., Dewaele S., Libert C., Engelborghs S., De Deyn P.P., Toussaint O., Debacq-Chainiaux F., Poulain M., Glupczynski Y., Franceschi C. (2010). Serum N-glycan profile shift during human ageing. Exp. Gerontol..

[12] Franceschi C., Garagnani P., Morsiani C., Conte M., Santoro A., Grignolio A., Monti D., Capri M., Salvioli S. (2018). The continuum of aging and age-related diseases: Common mechanisms but different rates. Front. Med..

[13] López-Otín C., Blasco M.A., Partridge L., Serrano M., Kroemer G. (2013). The hallmarks of aging Europe PMC funders group. Cell.

[14] Minuti A., Patrone V., Giuberti G., Spigno G., Pietri A., Battilani P., Ajmone Marsan P. (2014). Nutrition and ageing. Stud. Health Technol. Inform..

[15] Kim T., Xie Y., Li Q., Artegoitia V.M., Lebrilla C.B., Keim N.L., Adams S.H., Krishnan S. (2021). Diet affects glycosylation of serum proteins in women at risk for cardiometabolic disease. Eur. J. Nutr..

[16] Vreeker G.C.M., Wuhrer M. (2017). Reversed-phase separation methods for glycan analysis. Anal. Bioanal. Chem..

[17] Geyer H., Geyer R. (2006). Strategies for analysis of glycoprotein glycosylation. Biochim. Biophys. Acta Proteins Proteomics.

[18] Wuhrer M., Deelder A.M., Hokke C.H. (2005). Protein glycosylation analysis by liquid chromatography-mass spectrometry. J. Chromatogr. B Anal. Technol. Biomed. Life Sci..

[19] Testa R., Vanhooren V., Bonfigli A.R., Boemi M., Olivieri F., Ceriello A., Genovese S., Spazzafumo L., Borelli V., Bacalini M.G. (2015). N-Glycomic changes in serum proteins in type 2 diabetes mellitus correlate with complications and with metabolic syndrome parameters. PLoS ONE.

[20] Nilsson A., Santoro A., Franceschi C., Kadi F. (2019). Detrimental links between physical inactivity, metabolic risk and N-glycomic biomarkers of aging. Exp. Gerontol..

[21] Wang H., Li X., Wang X., Liu D., Zhang X., Cao W., Zheng Y., Guo Z., Li D., Xing W. (2019). Next-generation (glycomic) biomarkers for cardiometabolic health: A community-based study of immunoglobulin G N-glycans in a chinese han population. Omi. A J. Integr. Biol..

[22] Vanhooren V., Desmyter L., Liu X.E., Cardelli M., Franceschi C., Federico A., Libert C., Laroy W., Dewaele S., Contreras R. (2007). N-glycomic changes in serum proteins during human aging. Rejuvenation Res..

[23] Šimurina M., de Haan N., Vučković F., Kennedy N.A., Štambuk J., Falck D., Trbojević-Akmačić I., Clerc F., Razdorov G., Khon A. (2018). Glycosylation of immunoglobulin g associates with clinical features of inflammatory bowel diseases. Gastroenterology.

[24] Lundström S.L., Yang H., Lyutvinskiy Y., Rutishauser D., Herukka S.K., Soininen H., Zubarev R.A. (2014). Blood plasma IgG Fc glycans are significantly altered in Alzheimer’s disease and progressive mild cognitive impairment. J. Alzheimer Dis..

[25] Ge S., Wang Y., Song M., Li X., Yu X., Wang H., Wang J., Zeng Q., Wang W. (2018). Type 2 diabetes mellitus: Integrative analysis of multiomics data for biomarker discovery. Omi. A J. Integr. Biol..

[26] Kawaguchi-Sakita N., Kaneshiro-Nakagawa K., Kawashima M., Sugimoto M., Tokiwa M., Suzuki E., Kajihara S., Fujita Y., Iwamoto S., Tanaka K. (2016). Serum immunoglobulin G Fc region N-glycosylation profiling by matrix-assisted laser desorption/ionization mass spectrometry can distinguish breast cancer patients from cancer-free controls. Biochem. Biophys. Res. Commun..

[27] Leeflang B.R., Vliegenthart J.F.G. (2012). Glycoprotein analysis: Using nuclear magnetic resonance. Encycl. Anal. Chem..

[28] Harvey D.J. (2001). Identification of protein-bound carbohydrates by mass spectrometry. Proteomics.

[29] Mariño K., Bones J., Kattla J.J., Rudd P.M. (2010). A systematic approach to protein glycosylation analysis: A path through the maze. Nat. Chem. Biol..

[30] Jensen P.H., Kolarich D., Packer N.H. (2010). Mucin-type O-glycosylation—Putting the pieces together. FEBS J..

[31] Kennedy B.K., Berger S.L., Brunet A., Campisi J., Cuervo A.M., Epel E.S., Franceschi C., Lithgow G.J., Moritomo R.I., Pessin J.E. (2014). Aging: A common driver of chronic diseases and a target for novel interventions. Cell.

[32] Dall’Olio F., Vanhooren V., Chen C.C., Slagboom P.E., Wuhrer M., Franceschi C. (2013). N-glycomic biomarkers of biological aging and longevity: A link with inflammaging. Ageing Res. Rev..

[33] Everest-Dass A.V., Moh E.S.X., Ashwood C., Shathili A.M.M., Packer N.H. (2018). Human disease glycomics: Technology advances enabling protein glycosylation analysis–part 2. Expert Rev. Proteomics.

[34] Rudman N., Gornik O., Lauc G. (2019). Altered N-glycosylation profiles as potential biomarkers and drug targets in diabetes. FEBS Lett..

[35] Kizuka Y., Kitazume S., Taniguchi N. (2017). N-glycan and Alzheimer’s disease. Biochim. Biophys. Acta Gen. Subj..

[36] Konjevod M., Tudor L., Svob Strac D., Nedic Erjavec G., Barbas C., Zarkovic N., Nikolac Perkovic M., Uzun S., Kozumplik O., Lauc G. (2019). Metabolomic and glycomic findings in posttraumatic stress disorder. Prog. Neuro-Psychopharmacol. Biol. Psychiatry.

[37] Hu M., Lan Y., Lu A., Ma X., Zhang L. (2019). Progress in Molecular Biology and Translational Science.

[38] Zhang M., Dou H., Yang D., Shan M., Li X., Hao C., Zhang Y., Zeng P., He Y., Liu Y. (2019). Retrospective Analysis of Glycan-related Biomarkers Based on Clinical Laboratory Data in Two Medical Centers During the Past 6 Years. Prog Mol Biol Transl Sci.

[39] Nimmerjahn F., Anthony R.M., Ravetch J.V. (2007). Agalactosylated IgG antibodies depend on cellular Fc receptors for in vivo activity. Proc. Natl. Acad. Sci. USA.

[40] Russell A.C., Šimurina M., Garcia M.T., Novokmet M., Wang Y., Rudan I., Campbell H., Lauc G., Thomas M.G., Wang W. (2017). The N-glycosylation of immunoglobulin G as a novel biomarker of Parkinson’s disease. Glycobiology.

[41] Arnold J.N., Wormald M.R., Sim R.B., Rudd P.M., Dwek R.A. (2007). The impact of glycosylation on the biological function and structure of human immunoglobulins. Ann. Rev. Immunol..

[42] Shade K.-T., Anthony R. (2013). Antibody glycosylation and inflammation. Antibodies.

[43] Pučić M., Knežević A., Vidič J., Adamczyk B., Novokmet M., Polašek O., Gornik O., Šupraha-Goreta S., Wormald M.R., Redžic I. (2011). High throughput isolation and glycosylation analysis of IgG-variability and heritability of the IgG glycome in three isolated human populations. Mol. Cell. Proteomics.

[44] Parekh R., Isenberg D., Rook G., Roitt I., Dwek R., Rademacher T. (1989). A comparative analysis of disease-associated changes in the galactosylation of serum IgG. J. Autoimmun..

[45] Dotz V., Wuhrer M. (2019). N-glycome signatures in human plasma: Associations with physiology and major diseases. FEBS Lett..

[46] Fontana L., Partridge L., Longo V.D. (2010). Extending healthy life span—From yeast to humans. Science.

[47] Committee on Assessing the Importance and Impact of Glycomics and Glycosciences (2012). Transforming Glycoscience: A Roadmap for the Future.

[48] Krištić J., Vučković F., Menni C., Klarić L., Keser T., Beceheli I., Pučić-Baković M., Novokmet M., Mangino M., Thaqi K. (2014). Glycans are a novel biomarker of chronological and biological ages. J. Gerontol. Ser. A Biol. Sci. Med. Sci..

[49] Yu X., Wang Y., Kristic J., Dong J., Chu X., Ge S., Wang H., Fang H., Gao Q., Liu D. (2016). Profiling IgG N-glycans as potential biomarker of chronological and biological ages: A community-based study in a Han Chinese population. Medicine.

[50] Šunderić M., Križáková M., Malenković V., Ćujić D., Katrlík J., Nedić O. (2019). Changes due to ageing in the glycan structure of α-2-macroglobulin and its reactivity with ligands. Protein J..

[51] Calvert L., Atkinson H., Berry L., Chan A. (2019). Age-dependent variation in glycosylation features of α-2-macroglobulin. Cell Biochem. Biophys..

[52] Robajac D., Masnikosa R., Nemčovič M., Križáková M., Belická Kluková Ľ., Baráth P., Katrlík J., Nedić O. (2019). Glycoanalysis of the placental membrane glycoproteins throughout placental development. Mech. Ageing Dev..

[53] Wide L., Eriksson K. (2019). Unique pattern of N-glycosylation, sialylation, and sulfonation on TSH molecules in serum of children up to 18 months. J. Clin. Endocrinol. Metab..

[54] Donovan M., Bernard D., Simonetti L., Cavusoglu N., Rudd P., Duke R. (2019). Skin glycomics—Characterization of the N-glycome in the stratum corneum of aged and dry skin. J. Investig. Dermatol..

[55] Baković M.P., Selman M.H.J., Hoffmann M., Rudan I., Campbell H., Deelder A.M., Lauc G., Wuhrer M. (2013). High-throughput IgG Fc N-glycosylation profiling by mass spectrometry of glycopeptides. J. Proteome Res..

[56] Ruhaak L.R., Uh H.W., Beekman M., Koeleman C.A.M., Hokke C.H., Westendorp R.G.J., Wuhrer M., Houwing-Duistermaat J.J., Slagboom P.E., Deelder A.M. (2010). Decreased levels of bisecting GLcNAc glycoforms of IgG are associated with human longevity. PLoS ONE.

[57] Reiding K.R., Ruhaak L.R., Uh H., Bouhaddani S., Van Den Akker E.B., Plomp R., Mcdonnell L.A., Houwing-duistermaat J.J., Slagboom P.E., Beekman M. (2017). Human Plasma N-glycosylation as analyzed by matrix-assisted laser desorption/ionization-fourier transform ion cyclotron resonance-MS associates with markers of inflammation and metabolic health. Mol. Cell Proteomics.

[58] Ruhaak L.R., Koeleman C.A.M., Uh H.W., Stam J.C., van Heemst D., Maier A.B., Houwing-Duistermaat J.J., Hensbergen P.J., Slagboom P.E., Deelder A.M. (2013). Targeted biomarker discovery by high throughput glycosylation profiling of human plasma α1-antitrypsin and immunoglobulin A. PLoS ONE.

[59] Ding N., Sun H.N., Sun W., Qu Y., Liu X., Yao Y., Liang X., Chen C.C., Li Y. (2011). Human serum N-glycan profiles are age and sex dependent. Age Ageing.

[60] De Haan N., Reiding K.R., Driessen G., Van Der Burg M., Wuhrer M. (2016). Changes in healthy human IgG Fc-glycosylation after birth and during early childhood. J. Proteome Res..

[61] Berry L.R., Van Walderveen M.C., Atkinson H.M., Chan A.K.C. (2013). Comparison of N-linked glycosylation of protein C in newborns and adults. Carbohydr. Res..

[62] Edelberg J.M., Enghild J.J., Pizzo S.V., Gonzalez-Gronow M. (1990). Neonatal plasminogen displays altered cell surface binding and activation kinetics: Correlation with increased glycosylation of the protein. J. Clin. Investig..

[63] Kim B.S., Choi C.W., Shin H., Jin S.P., Bae J.S., Han M., Seo E.Y., Chun J., Chung J.H. (2019). Comparison of the gut microbiota of centenarians in longevity villages of South Korea with those of other age groups. J. Microbiol. Biotechnol..

[64] Wang W., Gopal S., Pocock R., Xiao Z. (2019). Glycan mimetics from natural products: New therapeutic opportunities for neurodegenerative disease. Molecules.

[65] (2020). Alzheimer’s Associaton Report. 2020 Alzheimer’s disease facts and figures. Alzheimer Dement..

[66] Duyckaerts C., Delatour B., Potier M.C. (2009). Classification and basic pathology of Alzheimer disease. Acta Neuropathol.

[67] Deane R., Yan S.D., Submamaryan R.K., Larue B., Jovanovic S., Hogg E., Welch D., Manness L., Lin C., Yu J. (2003). Rage mediates amyloid-β peptide transport across the blood-brain barrier and accumulation in brain. Nat. Med..

[68] Edri-Brami M., Rosental B., Hayoun D., Welt M., Rosen H., Wirguin I., Nefussy B., Drory V.E., Porgador A., Lichtenstein R.G. (2012). Glycans in sera of amyotrophic lateral sclerosis patients and their role in killing neuronal cells. PLoS ONE.

[69] Costa J., Streich L., Pinto S., Pronto-Laborinho A., Nimtz M., Conradt H.S., de Carvalho M. (2019). Exploring cerebrospinal fluid IgG N-glycosylation as potential biomarker for amyotrophic lateral sclerosis. Mol. Neurobiol..

[70] Gonçalves M., Tillack L., de Carvalho M., Pinto S., Conradt H.S., Costa J. (2015). Phosphoneurofilament heavy chain and N-glycomics from the cerebrospinal fluid in amyotrophic lateral sclerosis. Clin. Chim. Acta.

[71] Váradi C., Nehéz K., Hornyák O., Viskolcz B., Bones J. (2019). Serum N-glycosylation in Parkinson’s disease: A novel approach for potential alterations. Molecules.

[72] Kanninen K., Goldsteins G., Auriola S., Alafuzoff I., Koistinaho J. (2004). Glycosylation changes in Alzheimer’s disease as revealed by a proteomic approach. Neurosci. Lett..

[73] Hovden H., Frederiksen J.L., Pedersen S.W. (2013). Immune system alterations in amyotrophic lateral sclerosis. Acta Neurol. Scand..

[74] Vanhooren V., Liu X.E., Franceschi C., Gao C.F., Libert C., Contreras R., Chen C. (2009). N-glycan profiles as tools in diagnosis of hepatocellular carcinoma and prediction of healthy human ageing. Mech. Ageing Dev..

[75] Kyselova Z., Mechref Y., Kang P., Goetz J.A., Dobrolecki L.E., Sledge G.W., Schnaper L., Hickey R.J., Malkas L.H., Novotny M.V. (2008). Breast cancer diagnosis and prognosis through quantitative measurements of serum glycan profiles. Clin. Chem..

[76] Hamid U.M.A., Royle L., Saldova R., Radcliffe C.M., Harvey D.J., Storr S.J., Pardo M., Antrobus R., Chapman C.J., Zitzmann N. (2008). A strategy to reveal potential glycan markers from serum glycoproteins associated with breast cancer progression. Glycobiology.

[77] An H., Miyamoto S., Lancaster K.S., Kirmiz C., Li B., Lam K.S., Leiserowitz G.S., Lebrilla C.B. (2006). Profiling of glycans in serum for the discovery of potential biomarkers for ovarian cancer. J. Proteome Res..

[78] Alley R., Novotny M. (2010). Glycomic analysis of sialic acid linkages in glycans derived from blood serum glycoproteins. J. Proteome Res..

[79] Liu L., Yan B., Huang J., Gu Q., Wang L., Fang M., Jiao J., Yue X. (2013). The identification and characterization of novel N-glycan-based biomarkers in gastric cancer. PLoS ONE.

[80] Drabik A., Bodzon-Kulakowska A., Suder P., Silberring J., Kulig J., Sierzega M. (2017). Glycosylation changes in serum proteins identify patients with pancreatic cancer. J. Proteome Res..

[81] Hu Y., Ferdosi S., Kapuruge E.P., Diaz de Leon J.A., Stücker I., Radoï L., Guénel P., Borges C.R. (2019). Diagnostic and prognostic performance of blood plasma glycan features in the women epidemiology lung cancer (WELCA) Study. J. Proteome Res..

[82] Penezić A., Križakova M., Miljuš G., Katrlik J., Nedić O. (2019). Diagnostic potential of transferrin glycoforms—A lectin-based protein microarray approach. Proteomics Clin. Appl..

[83] Qiu Y., Patwa T.H., Xu L., Shedden K., Misek D.E., Jin G., Ruffin M.T., Turgeon D.K., Synal S., Marcon N. (2009). Plasma glycoprotein profiling for colorectal cancer biomarker identification by lectin glycoarray and lectin blot. J. Proteome Res..

[84] Zhao Y.P., Ruan C.P., Wang H., Hu Z.Q., Fang M., Gu X., Ji J., Zhao J.Y., Gao C.F. (2012). Identification and assessment of new biomarkers for colorectal cancer with serum N-glycan profiling. Cancer.

[85] Šunderić M., Šedivá A., Robajac D., Miljuš G., Gemeiner P., Nedić O., Katrlík J. (2016). Lectin-based protein microarray analysis of differences in serum alpha-2-macroglobulin glycosylation between patients with colorectal cancer and persons without cancer. Biotechnol. Appl. Biochem..

[86] De Leoz M.L.A., An H.J., Kronewitter S., Kim J., Beecroft S., Vinall R., Miyamoto S., De Vere White R., Lam K.S., Lebrilla C. (2008). Glycomic approach for potential biomarkers on prostate cancer: Profiling of N-linked glycans in human sera and pRNS cell lines. Dis. Markers.

[87] Bereman M.S., Williams T.I., Muddiman D.C. (2009). Development of a nanolc ltq orbitrap mass spectrometric method for profiling glycans derived from plasma from healthy, benign tumor control, and epithelial ovarian cancer patients. Anal. Chem..

[88] Takahashi S., Sugiyama T., Shimomura M., Kamada Y., Fujita K., Nonomura N., Miyoshi E., Nakano M. (2016). Site-specific and linkage analyses of fucosylated N-glycans on haptoglobin in sera of patients with various types of cancer: Possible implication for the differential diagnosis of cancer. Glycoconj. J..

[89] Miyoshi E., Moriwaki K., Nakagawa T. (2008). Biological function of fucosylation in cancer biology. J. Biochem..

[90] Li Q., Li G., Zhou Y., Zhang X., Sun M., Jiang H., Yu G. (2019). Comprehensive N-glycome profiling of cells and tissues for breast cancer diagnosis. J. Proteome Res..

[91] Adua E., Memarian E., Russell A., Trbojević-Akmačić I., Gudelj I., Jurić J., Roberts P., Lauc G., Wang W. (2019). High throughput profiling of whole plasma N-glycans in type II diabetes mellitus patients and healthy individuals: A perspective from a Ghanaian population. Arch. Biochem. Biophys..

[92] Dotz V., Lemmers R.F.H., Reiding K.R., Hipgrave Ederveen A.L., Lieverse A.G., Mulder M.T., Sijbrands E.J.G., Wuhrer M., van Hoek M. (2018). Plasma protein N-glycan signatures of type 2 diabetes. Biochim. Biophys. Acta Gen. Subj..

[93] Connelly M.A., Gruppen E.G., Wolak-Dinsmore J., Matyus S.P., Riphagen I.J., Shalaurova I., Bakker S.J.L., Otvos J.D., Dullaart R.P.F. (2016). GlycA, a marker of acute phase glycoproteins, and the risk of incident type 2 diabetes mellitus: Prevend study. Clin. Chim. Acta.

[94] Grundy S.M., Hansen B., Smith S.C., Cleeman J.I., Kahn R.A. (2004). Clinical management of metabolic syndrome. Arterioscler. Thromb. Vasc. Biol..

[95] Lu J.P., Knežević A., Wang Y.X., Rudan I., Campbell H., Zou Z.K., Lan J., Lai Q.X., Wu J.J., He Y. (2011). Screening novel biomarkers for metabolic syndrome by profiling human plasma N-glycans in Chinese Han and Croatian populations. J. Proteome Res..

[96] Gao Q., Dolikun M., Štambuk J., Wang H., Zhao F., Yiliham N., Wang Y., Trbojević-Akmačić I., Zhang J., Fang H. (2017). immunoglobulin G N-glycans as potential postgenomic biomarkers for hypertension in the Kazakh population. Omi. A J. Integr. Biol..

[97] Wang Y., Klarić L., Yu X., Thaqi K., Dong J., Novokmet M., Wilson J., Polasek O., Liu Y., Krištić J. (2016). The association between glycosylation of immunoglobulin G and hypertension. Medicine.

[98] Liu J., Dolikun M., Štambuk J., Trbojević-Akmačić I., Zhang J., Wang H., Zheng D., Zhang X., Peng H., Zhao Z. (2018). The association between subclass-specific IgG Fc N-glycosylation profiles and hypertension in the Uygur, Kazak, Kirgiz, and Tajik populations. J. Hum. Hypertens..

[99] Menni C., Gudelj I., MacDonald-Dunlop E., Mangino M., Zierer J., Bešić E., Joshi P.K., Trbojević-Akmačić I., Chowienczyk P.J., Spector T.D. (2018). Glycosylation profile of immunoglobulin g is cross-sectionally associated with cardiovascular disease risk score and subclinical atherosclerosis in two independent cohorts. Circ. Res..

[100] Barrios C., Zierer J., Gudelj I., Stambuk J., Ugrina I., Rodríguez E., Soler M.J., Pavic T., Simurina M., Keser T. (2016). Glycosylation profile of IgG in moderate kidney dysfunction. J. Am. Soc. Nephrol..

[101] Liu D., Chu X., Wang H., Dong J., Ge S.Q., Zhao Z.Y., Peng H.L., Sun M., Wu L.J., Song M.S. (2018). The changes of immunoglobulin G N-glycosylation in blood lipids and dyslipidaemia. J. Transl. Med..

[102] Wilson P.W.F., D’Agostino R.B., Parise H., Sullivan L., Meigs J.B. (2005). Metabolic syndrome as a precursor of cardiovascular disease and type 2 diabetes mellitus. Circulation.

[103] North B.J., David A. (2012). Sinclair the intersection between aging and cardiovascular disease. Circ. Res..

[104] Vasudevan A.R., Ballantyne C.M. (2005). Cardiometabolic risk assessment: An approach to the prevention of cardiovascular disease and diabetes mellitus. Clin. Cornerstone.

[105] Umaña P., Jean-Mairet J., Moudry R., Amstutz H., Bailey J.E. (1999). Engineered glycoforms of an antineuroblastoma IgG1 with optimized antibody-dependent cellular cytotoxic activity. Nat. Biotechnol..

[106] Willerson J.T., Ridker P.M. (2004). Inflammation as a cardiovascular risk factor. Circulation.

[107] Böhm S., Schwab I., Lux A., Nimmerjahn F. (2012). The role of sialic acid as a modulator of the anti-inflammatory activity of IgG. Semin. Immunopathol..

[108] Tenenbaum A., Klempfner R., Fisman E.Z. (2014). Hypertriglyceridemia: A too long unfairly neglected major cardiovascular risk factor. Cardiovasc. Diabetol..

[109] Buford T.W. (2016). Hypertension and aging. Ageing Res. Rev..

[110] Trbojevic Akmacic I., Ventham N.T., Theodoratou E., Vučković F., Kennedy N.A., Krištić J., Nimmo E.R., Kalla R., Drummond H., Štambuk J. (2015). Inflammatory bowel disease associates with proinflammatory potential of the immunoglobulin G glycome. Inflamm. Bowel Dis..

[111] El Nahas A.M., Bello A.K. (2005). Chronic kidney disease: The global challenge. Lancet.

[112] Clerc F., Novokmet M., Dotz V., Reiding K.R., de Haan N., Kammeijer G.S.M., Dalebout H., Bladergroen M.R., Vukovic F., Rapp E. (2018). Plasma N-glycan signatures are associated with features of inflammatory bowel diseases. Gastroenterology.

[113] Schultz M.J., Swindall A.F., Bellis S.L. (2012). Regulation of the metastatic cell phenotype by sialylated glycans. Cancer Metastasis Rev..

[114] Dalziel M., Crispin M., Scanlan C.N., Zitzmann N., Dwek R.A. (2014). Emerging principles for the therapeutic exploitation of glycosylation. Science.

[115] Shinzaki S., Iijima H., Nakagawa T., Egawa S., Nakajima S., Ishii S., Irie T., Kakiuchi Y., Nishida T., Yasumaru M. (2008). IgG oligosaccharide alterations are a novel diagnostic marker for disease activity and the clinical course of inflammatory bowel disease. Am. J. Gastroenterol..

[116] Pasek M., Duk M., Podbielska M., Sokolik R., Szechiński J., Lisowska E., Krotkiewski H. (2006). Galactosylation of IgG from rheumatoid arthritis (RA) patients—Changes during therapy. Glycoconj. J..

[117] Gudelj I., Salo P.P., Trbojević-Akmačić I., Albers M., Primorac D., Perola M., Lauc G. (2018). Low galactosylation of IgG associates with higher risk for future diagnosis of rheumatoid arthritis during 10 years of follow-up. Biochim. Biophys. Acta Mol. Basis Dis..

[118] Magorivska I., Döncző B., Dumych T., Karmash A., Boichuk M., Hychka K., Mihalj M., Szabó M., Csánky E., Rech J. (2018). Glycosylation of random IgG distinguishes seropositive and seronegative rheumatoid arthritis. Autoimmunity.

[119] Ercan A., Cui J., Chatterton D.E.W., Deane K.D., Hazen M.M., Brintnell W., Donnell C.I.O., Derber L.A., Weinblatt M.E., Nancy A. (2010). IgG galactosylation aberrancy precedes disease onset, correlates with disease activity and is prevalent in autoantibodies in rheumatoid arthritis. Arthritis Rheum..

[120] Gindzienska-Sieskiewicz E., Klimiuk P.A., Kisiel D.G., Gindzienski A., Sierakowski S. (2007). The changes in monosaccharide composition of immunoglobulin G in the course of rheumatoid arthritis. Clin. Rheumatol..

[121] Sebastian A., Alzain M.A., Asweto C.O., Song H., Cui L., Yu X., Ge S., Dong H., Rao P., Wang H. (2016). Glycan biomarkers for rheumatoid arthritis and its remission status in Han Chinese patients. Omi. A J. Integr. Biol..

[122] Huffman J.E., Pučić-Baković M., Klarić L., Hennig R., Selman M.H.J., Vučković F., Novokmet M., Krištić J., Borowiak M., Muth T. (2014). Comparative performance of four methods for high-throughput glycosylation analysis of immunoglobulin G in genetic and epidemiological research. Mol. Cell. Proteomics.

[123] Fernandes-Cerqueira C., Renard N., Notarnicola A., Wigren E., Gräslund S., Zubarev R.A., Lundberg I.E., Lundström S.L. (2018). Patients with anti-Jo1 antibodies display a characteristic IgG Fc-glycan profile which is further enhanced in anti-Jo1 autoantibodies. Sci. Rep..

[124] Lee D.M., Weinblatt M.E. (2001). Rheumatoid arthritis. Lancet..

[125] Mastrangelo A., Colasanti T., Barbati C., Pecani A., Sabatinelli D., Pendolino M., Truglia S., Massaro L., Mancini R., Miranda F. (2015). The role of posttranslational protein modifications in rheumatological diseases: Focus on rheumatoid arthritis. J. Immunol. Res..

[126] Albrecht S., Unwin L., Muniyappa M., Rudd P.M. (2014). Glycosylation as a marker for inflammatory arthritis. Cancer Biomarkers.

[127] Parekh R.B., Dwek R.A., Sutton B.J., Fernandes D.L., Leung A., Stanworth D., Rademacher T.W., Mizuochi T., Taniguchi T., Matsuta K. (1985). Association of rheumatoid arthritis and primary osteoarthritis with changes in the glycosylation pattern of total serum IgG. Nature.

[128] Johnson C., Pinal-Fernandez I., Parikh R., Paik J., Albayda J., Mammen A.L., Christopher-Stine L., Danoff S. (2016). Assessment of mortality in autoimmune myositis with and without associated interstitial lung disease. Lung.

[129] Han L., Costello C. (2013). Mass spectrometry of glycans. Biochemistry.

[130] Hirabayashi J., Yamada M., Kuno A., Tateno H. (2013). Lectin microarrays: Concept, principle and applications. Chem. Soc. Rev..

[131] Zhang L., Luo S., Zhang B. (2016). The use of lectin microarray for assessing glycosylation of therapeutic proteins. MAbs.

[132] Zhang Y., Peng Y., Yang L., Lu H. (2017). Advances in sample preparation strategies for MS-based qualitative and quantitative N-Glycomics. Trends Anal. Chem..

[133] Xiao K., Han Y., Yang H., Lu H., Tian Z. (2019). Mass spectrometry-based qualitative and quantitative N-glycomics: An update of 2017–2018. Anal. Chim. Acta.

[134] Takasaki S., Mizuochi T., Kobata A. (1982). Hydrazinolysis of asparagine-linked sugar chains to produce free oligosaccharides. Methods Enzymol..

[135] Rasilo M., Renkonen O. (1981). Mild alkaline borohydride treatment liberates N-acetylglucosamine linked oligosaccharide chains of glycoproteins. FEBS Lett..

[136] Maley F., Trimble R.B., Tarentino A.L., Plummer T.H. (1989). Characterization of glycoproteins and their associated oligosaccharides through the use of endoglycosidases. Anal. Biochem..

[137] Lauber M.A., Yu Y., Brousmiche D.W., Hua Z., Koza S.M., Magnelli P., Guthrie E., Taron C.H., Fountain K.J. (2015). Rapid preparation of released N-glycans for HILIC analysis using a labeling reagent that facilitates sensitive fluorescence and ESI-MS detection. Anal. Chem..

[138] Sandoval W.N., Arellano F., Arnott D., Raab H., Vandlen R., Lill J.R. (2007). Rapid removal of N-linked oligosaccharides using microwave assisted enzyme catalyzed deglycosylation. Int. J. Mass Spec..

[139] Szabo Z., Karger B.L. (2010). Rapid release of N-linked glycans from glycoproteins by pressure-cycling technology. Anal. Chem..

[140] Palm A.K., Novotny M.V. (2005). A monolithic PNGase F enzyme microreactor enabling glycan mass mapping of glycoproteins by mass spectrometry. Rapid Commun Mass Spectrom..

[141] Song T., Aldredge D., Lebrilla C.B. (2017). A Method for in-depth structural annotation of human serum glycans that yields biological variations. Anal. Chem..

[142] Fanayan S., Hincapie M., Hancock W.S. (2012). Using lectins to harvest the plasma/serum glycoproteome. Electrophoresis.

[143] Etxebarria J., Calvo J., Martin-Lomas M., Reichardt N.C. (2012). Lectin-array blotting: Profiling protein glycosylation in complex mixtures. ACS Chem. Biol..

[144] Ribeiro J.P., Mahal L.K. (2013). Dot by dot: Analyzing the glycome using lectin microarrays João. Curr. Opin. Chem. Biol..

[145] Keser T., Pavić T., Lauc G., Gornik O. (2018). Comparison of 2-aminobenzamide, procainamide and RapiFluor-MS as derivatizing agents for high-throughput HILIC-UPLC-FLR-MS N-glycan analysis. Front. Chem..

[146] Alpert A.J. (1990). Hydrophilic-interaction chromatography for the separation of peptides, nucleic acids and other polar compounds. J. Chromatogr. A.

[147] Takegawa Y., Deguchi K., Ito H., Keira T., Nakagawa H., Nishimura S.I. (2006). Simple separation of isometric sialylated N-glycopeptides by a zwitterionic type of hydrophilic interaction chromatography. J. Sep. Sci..

[148] Buszewski B., Noga S. (2012). Hydrophilic interaction liquid chromatography (HILIC)-a powerful separation technique. Anal. Bioanal. Chem..

[149] Wuhrer M., Boer A.R., Deelder A. (2009). Structural glycomics using hydrophilic interaction chromatography (HILIC) with mass spectrometry. Mass Spectrom. Rev..

[150] Takahashi N. (1996). Three-dimensional mapping of N-linked oligosaccharides using anion-exchange, hydrophobic and hydrophilic interaction modes of high-performance liquid chromatography. J. Chromatogr. A.

[151] El Rassi Z. (1994). Carbohydrate Analysis: High Performance Liquid Chromatography and Capillary Electrophoresis.

[152] Zhou S., Veillon L., Dong X., Huang Y., Mechref Y. (2017). Direct comparison of derivatization strategies for LC-MS/MS analysis of N-glycans. Analyst.

[153] Ruhaak L.R., Deelder A.M., Wuhrer M., Zauner G., Bruggink C., Huhn C. (2010). Glycan labeling strategies and their use in identification and quantification. Anal. Bioanal. Chem..

[154] Cohen S.A., Michaud D.P. (1993). Synthesis of a fluorescent derivatizing reagent,6-aminoquinolyl-N-hydroxysuccinimidyl carbamate, and its application forthe analysis of hydrolysate amino acids via high-performance liquidchromatography. Anal. Biochem..

[155] Wu Y., Sha Q., Wang C., Liu B., Wang S., Liu X. (2019). Development of a filter-aided extraction method coupled with glycosylamine labeling to simplify and enhance high performance liquid chromatography-based N-glycan analysis. J. Chromatogr. A.

[156] Lu G., Crihfield C.L., Gattu S., Veltri L.M., Holland L.A. (2018). Capillary electrophoresis separations of glycans. Chem. Rev..

[157] Donczo B., Szarka M., Tovari J., Ostoros G., Csanky E., Guttman A. (2017). Molecular glycopathology by capillary electrophoresis: Analysis of the N-glycome of formalin-fixed paraffin-embedded mouse tissue samples. Electrophoresis.

[158] Guttman A. (1996). High-resolution carbohydrate profiling by capillary gel electrophoresis. Nature.

[159] Mahan A.E., Tedesco J., Dionne K., Baruah K., Cheng H.D., De Jager P.L., Barouch D.H., Suscovich T., Ackerman M., Cripsin M. (2015). A method for high-throughput, sensitive analysis of IgG Fc and Fab glycosylation by capillary electrophoresis. J. Immunol. Methods.

[160] Reusch D., Haberger M., Kailich T., Heidenreich A.K., Kampe M., Bulau P., Wuhrer M. (2014). High-throughput glycosylation analysis of therapeutic immunoglobulin G by capillary gel electrophoresis using a DNA analyzer. MAbs.

[161] Adamczyk B., Tharmalingam-Jaikaran T., Schomberg M., Szekrényes Á., Kelly R.M., Karlsson N.G., Guttman A., Rudd P.M. (2014). Comparison of separation techniques for the elucidation of IgG N-glycans pooled from healthy mammalian species. Carbohydr. Res..

[162] Vanhooren V., Laroy W., Libert C., Chen C. (2008). N-Glycan profiling in the study of human aging. Biogerontology.

[163] Echeverria B., Etxebarria J., Ruiz N., Hernandez Á., Calvo J., Haberger M., Reusch D., Reichardt N.C. (2015). Chemo-enzymatic synthesis of 13C labeled complex N-glycans as internal standards for the absolute glycan quantification by mass spectrometry. Anal. Chem..

[164] Smith J., Mittermayr S., Váradi C., Bones J. (2017). Quantitative glycomics using liquid phase separations coupled to mass spectrometry. Analyst.

[165] Chen J.E., Glover G.H. (2016). Functional magnetic resonance imaging methods. Neuropsychol. Rev..

[166] Zhou S., Hu Y., DeSantos-Garcia J.L., Mechref Y. (2015). Quantitation of permethylated N-glycans through multiple-reaction monitoring (MRM) LC-MS/MS. J. Am. Soc. Mass Spectrom..

[167] Wu S., Salcedo J., Tang N., Waddell K., Grimm R., German J.B., Lebrilla C.B. (2012). Employment of tandem mass spectrometry for the accurate and specific identification of oligosaccharide structures. Anal. Chem..

[168] Sandra K., Devreese B., Van Beeumen J., Stals I., Claeyssens M. (2004). The Q-Trap mass spectrometer, a novel tool in the study of protein glycosylation. J. Am. Soc. Mass Spectrom..

[169] Zhou W., Hakansson K. (2011). Structural characterization of carbohydrates by fourier transform tandem mass spectrometry. Curr. Proteomics.

[170] Mechref Y., Novotny M.V., Krishnan C. (2003). Structural characterization of oligosaccharides using MALDI-TOF/TOF tandem mass spectrometry. Anal. Chem..

[171] Leymarie N., Zaia J. (2012). Effective use of mass spectrometry for glycan and glycopeptide structural analysis. Anal. Chem..

[172] Dell A. (2002). Glycoprotein structure determination by mass spectrometry. Science.

[173] Wang C., Li J., Yao S., Guo Y., Xia X. (2007). High-sensitivity matrix-assisted laser desorption/ionization Fourier transform mass spectrometry analyses of small carbohydrates and amino acids using oxidized carbon nanotubes prepared by chemical vapor deposition as matrix. Anal. Chim. Acta.

[174] Harvey D.J. (2012). Analysis of carbohydrates and glycoconjugates by matrix-assisted laser desorption/ionization mass spectrometry: An update for 2007–2008. Mass Spectrom. Rev..

[175] Borowsky A.D., Clowers B.H., Lebrilla C.B., Miyamoto S., Ferrige A., An H.J., Lam K.S., Kirmiz C., Li B., Alecio R. (2006). A serum glycomics approach to breast cancer biomarkers. Mol. Cell. Proteomics.

[176] Huang C., Yan J., Zhan L., Zhao M., Zhou J. (2019). Linkage and sequence analysis of neutral oligosaccharides by negative-ion MALDI tandem mass spectrometry with laser-induced dissociation. Anal. Chim. Acta.

[177] Briggs M.T., Kuliwaba J.S., Muratovic D., Everest-Dass A.V., Packer N.H., Findlay D.M., Hoffmann P. (2016). MALDI mass spectrometry imaging of N-glycans on tibial cartilage and subchondral bone proteins in knee osteoarthritis. Proteomics.

[178] Angel P.M., Mehta A., Norris-Caneda K., Drake R.R. (2018). MALDI imaging mass spectrometry of N-glycans and tryptic peptides from the same formalin-fixed, paraffin-embedded tissue section. Methods Mol. Biol..

[179] Zhou S., Wooding K., Mechref Y. (2017). Analysis of permethylated glycan by liquid chromatography (LC) and mass spectrometry (MS). Methods Mol. Biol. Glycomics Methods Protoc..

[180] Grünwald-Gruber C., Thader A., Maresch D., Dalik T., Altmann F. (2017). Determination of true ratios of different N-glycan structures in electrospray ionization mass spectrometry. Anal. Bioanal. Chem..

[181] Bruins A.P. (1998). Mechanistic aspects of electrospray ionization. J. Chromatogr. A.

[182] Wuhrer M., Koeleman C.A., Deelder A.M. (2009). Two-Dimensional HPLC Separation with reverse-phase-nano-LC-MS/MS for the characterization of glycan pools after labeling with 2-aminobenzamide. Glycomics Methods Protoc..

[183] Wuhrer M., Koeleman C.A.M., Fitzpatrick J.M., Hoffmann K.F., Deelder A.M., Hokke C.H. (2006). Gender-specific expression of complex-type N-glycans in schistosomes. Glycobiology.

[184] Kozak R.P., Tortosa C.B., Fernandes D.L., Spencer D.I.R. (2015). Comparison of procainamide and 2-aminobenzamide labeling for profiling and identification of glycans liquid chromatography with fluorescence detection coupled to electrospray ionization-mass spectrometry. Anal. Biochem..

[185] Li B., An H.J., Hedrick J.L., Lebrilla C.B. (2009). Collision-induced dissociation tandem mass spectrometry for structural elucidation of glycans. Methods Mol. Biol. Glycomics Methods Protoc..

[186] Sleno L., Volmer D.A. (2004). Ion activation methods for tandem mass spectrometry. J. Mass Spectrom..

[187] Domon B., Costello C.E. (1988). A systematic nomenclature for carbohydrate fragmentations in FAB-MS/MS spectra of glycoconjugates. Glycoconj. J..

[188] Zhou S., Dong X., Veillon L., Huang Y., Mechref Y. (2017). LC-MS/MS analysis of permethylated N-glycans facilitating isomeric characterization. Anal. Bioanal Chem..

[189] Wang P. (2005). Altered glycosylation in cancer: Sialic acids and sialyltransferases. J. Cancer Mol..

[190] Wada Y., Azadi P., Costello C.E., Dell A., Dwek R.A., Geyer H., Geyer R., Kakehi K., Karlsson N.G., Kato K. (2007). Comparison of the methods for profiling glycoprotein glycans—HUPO human disease glycomics/proteome initiative multi-institutional study. Glycobiology.

[191] Mechref Y., Hu Y., Desantos-Garcia J.L., Hussein A., Tang H. (2013). Quantitative glycomics strategies. Mol. Cell. Proteomics.

[192] Hu Y., Borges C.R. (2017). A spin column-free approach to sodium hydroxide-based glycan permethylation. Analyst.

[193] Powell A.K., Harvey D.J. (1996). Stabilization of sialic acids in N-linked oligosaccharides and gangliosides for analysis by positive ion matrix-assisted laser desorption/ionization mass spectrometry. Rapid Commun. Mass Spectrom..

[194] Kita Y., Miura Y., Furukawa J., Nakano M., Shinohara Y., Ohno M., Takimoto A., Nishimura S.I. (2007). Quantitative glycomics of human whole serum glycoproteins based on the standardized protocol for liberating N-glycans. Mol. Cell. Proteomics.

[195] Wheeler S.F., Domann P., Harvey D.J. (2009). Derivatization of sialic acids for stabilization in matrix-assisted laser desorption/ionization mass spectrometry and concomitant differentiation of α(2 --> 3)- and α(2 --> 6)-isomers. Rapid Commun. Mass Spectrom..

[196] Reiding K.R., Blank D., Kuijper D.M., Deelder A.M., Wuhrer M. (2014). High-throughput profiling of protein N-glycosylation by MALDI-TOF-MS employing linkage-specific sialic acid esterification. Anal. Chem..

[197] Toyoda M., Ito H., Matsuno Y.K., Narimatsu H., Kameyama A. (2008). Quantitative derivatization of sialic acids for the detection of sialoglycans by MALDI MS. Anal. Chem..

[198] Gil G., Iliff B., Cerny R., Velander W.H., Van Cott K.E. (2010). High throughput quantification of N-glycans using one-pot sialic acid modification and matrix assisted laser desorption ionization time of flight mass spectrometry. Anal. Chem..

[199] De Haan N., Reiding K.R., Haberger M., Reusch D., Falck D., Wuhrer M. (2015). Linkage-specific sialic acid derivatization for MALDI-TOF-MS profiling of IgG glycopeptides. Anal. Chem..

[200] Sekiya S., Wada Y., Tanaka K. (2005). Derivatization for stabilizing sialic acids in MALDI-MS. Society.

[201] Cummings R.D., Pierce J.M. (2009). Handbook of Glycomics.

[202] Kailemia M.J., Ruhaak L.R., Lebrilla C.B., Amster I.J. (2015). Oligosaccharide analysis by mass spectrometry: A review of recent developments. Anal. Chem..

[203] Kohler J.J., Patrie S.M. (2013). Mass Spectrometry of Glycoproteins: Methods and Protocols.

[204] Kang P., Mechref Y., Kyselova Z., Goetz J.A., Novotny M.V. (2007). Comparative glycomic mapping through quantitative permethylation and stable-isotope labeling. Anal. Chem..

[205] Ma H., Miao X., Ma Q., Zheng W., Zhou H., Jia L. (2013). Functional roles of glycogene and N-glycan in multidrug resistance of human breast cancer cells. IUBMB Life.

[206] Atwood J.A., Cheng L., Alvarez-manilla G., Warren N.L., York W.S., Orlando R. (2008). Quantitation by isobaric labeling: Applications to glycomics James. J. Proteome Res..

[207] Apte A., Meitei N.S. (2010). Bioinformatics in glycomics: Glycan characterization with mass spectrometric data using SimGlycan. Methods Mol. Biol..

[208] Morrison K.A., Clowers B.H. (2018). Contemporary glycomic approaches using ion mobility–mass spectrometry. Curr. Opin. Chem. Biol..

[209] Barroso A., Giménez E., Konijnenberg A., Sancho J., Sanz-Nebot V., Sobott F. (2018). Evaluation of ion mobility for the separation of glycoconjugate isomers due to different types of sialic acid linkage, at the intact glycoprotein, glycopeptide and glycan level. J. Proteomics.

[210] Ruotolo B.T., Benesch J.L.P., Sandercock A.M., Hyung S.J., Robinson C.V. (2008). Ion mobility-mass spectrometry analysis of large protein complexes. Nat. Protoc..

[211] Bush M.F., Hall Z., Giles K., Hoyes J., Robinson C.V., Ruotolo B.T. (2010). Collision cross sections of proteins and their complexes: A calibration framework and database for gas-phase structural biology. Anal. Chem..

[212] Campbell M.P., Royle L., Radcliffe C.M., Dwek R.A., Rudd P.M. (2008). GlycoBase and autoGU: Tools for HPLC-based glycan analysis. Bioinformatics.

[213] Deshpande N., Jensen P.H., Packer N.H., Kolarich D. (2010). GlycoSpectrumScan: Fishing glycopeptides from MS spectra of protease digests of human colostrum sIgA research articles. J. Proteome Res..

[214] Lohmann K.K., von der Lieth C.W. (2004). GlycoFragment and GlycoSearchMS: Web tools to support the interpretation of mass spectra of complex carbohydrates. Nucleic Acids Res..

[215] Fellenberg M., Behnken H.N., Nagel T., Wiegandt A., Baerenfaenger M., Meyer B. (2013). Glycan analysis: Scope and limitations of different techniques—A case for integrated use of LC-MS(/MS) and NMR techniques. Anal. Bioanal. Chem..

[216] Hizal D.B., Wolozny D., Colao J., Jacobson E., Tian Y., Krag S.S., Betenbaugh M.J., Zhang H. (2014). Glycoproteomic and glycomic databases. Clin. Proteomics.

[217] Functional Glycomics Gateway. http://www.functionalglycomics.org/.

[218] Ranzinger R., Herget S., Wetter T., Von der Lieth W. (2008). GlycomeDB—Integration of open-access carbohydrate structure databases. BMC Bioinform..

[219] Cooper C.A., Joshi H.J., Harrison M.J., Wilkins M.R., Packer N.H. (2003). GlycoSuiteDB: A curated relational database of glycoprotein glycan structures and their biological sources. Nucleic Acids Res..

[220] Von Der Lieth C.W., Freire A.A., Blank D., Campbell M.P., Ceroni A., Damerell D.R., Dell A., Dwek R.A., Ernst B., Fogh R. (2011). EUROCarbDB: An open-access platform for glycoinformatics. Glycobiology.

[221] Hirabayashi J., Tateno H., Shikanai T., Aoki-Kinoshita K.F., Narimatsu H. (2015). The lectin frontier database (LfDB), and data generation based on frontal affinity chromatography. Molecules.

[222] Johannes F.G., Vliegenthart L.D., van Halbeek H. (1983). High-resolution, 1H-nuclear magnetic resonance spectrometry as a tool in the structural analysis of carbohydrates related to glycoproteins. Adv. Carbohydr. Chem. Biochem..

[223] Kam R.K.T., Poon T.C.W. (2008). The potentials of glycomics in biomarker discovery. Clin. Proteomics.

